# Microbiome and One Health in GCC countries: current status, research gaps, and future directions

**DOI:** 10.3389/fmicb.2026.1821688

**Published:** 2026-05-29

**Authors:** Marwh G. Aldriwesh, Hajer Bin Shuraym, Nouf Y. Asiri, Wejdan Y. Asiri, Norah F. Abukhalid, Abdulrahman Alasiri, Majed F. Alghoribi

**Affiliations:** 1 Department of Clinical Laboratory Sciences, College of Applied Medical Sciences, King Saud bin Abdulaziz University for Health Sciences, Riyadh, Saudi Arabia; 2 Department of Infectious Diseases, King Abdullah International Medical Research Center, Riyadh, Saudi Arabia; 3 Ministry of National Guard-Health Affairs, Riyadh, Saudi Arabia; 4 King Abdullah International Medical Research Center, Riyadh, Saudi Arabia; 5 Division of Microbiology, Department of Pathology and Laboratory Medicine, Ministry of the National Guard Health Affairs, Riyadh, Saudi Arabia; 6 Department of Anesthesia Technology, College of Applied Medical Sciences, King Saud bin Abdulaziz University for Health Sciences, Riyadh, Saudi Arabia; 7 Department of Cardiac Ancillary Services, King Faisal Specialist Hospital and Research Centre, Riyadh, Saudi Arabia; 8 Department of Artificial Intelligence and Data Management, Bioinformatics Division, King Abdullah International Medical Research Center, King Saud bin Abdulaziz University for Health Sciences, Riyadh, Saudi Arabia; 9 Department of Basic Science, College of Science and Health Professions, King Saud bin Abdulaziz University for Health Sciences, Riyadh, Saudi Arabia

**Keywords:** biotechnology, GCC, Gulf Cooperation Council, microbial ecology, microbiome, One Health, systematic review

## Abstract

**Background:**

Microbiome science has emerged as a central component of the One Health framework, linking human, animal, and environmental health. Although global microbiome research has expanded rapidly, a comprehensive evaluation of microbiome research development and integration across the Gulf Cooperation Council (GCC) countries remains lacking. This systematic review aimed to characterize microbiome research in the GCC countries, identify major research gaps, and evaluate alignment with One Health principles while proposing a strategic framework to support coordinated regional development.

**Methods:**

This systematic review followed PRISMA 2020 guidelines. A structured search of PubMed, ScienceDirect, Google Scholar, and EBSCO databases identified microbiome-related studies published up to January 31, 2025. Eligible studies included original research conducted in the GCC countries (Saudi Arabia, Qatar, Kuwait, United Arab Emirates, Oman, and Bahrain) investigating human, animal, or environmental microbiomes. Findings were synthesized descriptively to assess study distribution, research design, analytical methodologies, and thematic focus.

**Results:**

A total of 110 studies met the inclusion criteria. Human microbiome studies accounted for 49% of publications, followed by environmental microbiome studies (40%) and animal microbiome studies (11%). Research output increased substantially after 2020 but remained uneven among the GCC countries, with Saudi Arabia contributing 44% of publications, whereas Bahrain and Oman together accounted for fewer than 7%. Most studies were observational and primarily used 16S rRNA gene sequencing on Illumina platforms. Human studies focused mainly on gut and oral microbiomes and frequently investigated metabolic disorders such as obesity and diabetes. Animal microbiome research was limited and largely centered on camels, with minimal investigation of livestock relevant to food security. Environmental studies predominantly examined soil and desert environments. No included study simultaneously investigated human, animal, and environmental microbiomes within an integrated One Health study design.

**Conclusion:**

Microbiome research in the GCC countries is growing but remains uneven and largely disconnected across human, animal, and environmental studies, with limited adoption of One Health approaches. A coordinated regional strategy integrating governance, infrastructure, funding, and workforce development is needed to advance translational microbiome research and strengthen the GCC's contribution to global health, food security, and environmental sustainability.

## 1 Introduction

The term *microbiome* refers to the collective genomes of microorganisms inhabiting a specific environment (Ma et al., 2023). The microbiome comprises bacteria, archaea, eukaryotes, and viruses that colonize various body sites, including the skin, gastrointestinal tract, respiratory system, reproductive organs, and urinary tract (Govender and Ghai, 2025). It plays a vital role in multiple physiological processes, including digestion, immune modulation, neural regulation, and metabolic homeostasis across all stages of life, from early development to aging. It also contributes to postmortem processes, including decomposition of the body (Ma et al., 2024).

The microbiome also plays a critical role in animal health and fitness, influencing disease susceptibility, body condition, survival, and reproductive success (Worsley et al., 2024). Elements of microbial communities can be exchanged between humans and animals through close contact. Studies have shown that close physical interactions, particularly between humans and their pets, are associated with increased skin microbiome diversity and microbial sharing (Song et al., 2013; Ross et al., 2017). Human and animal microbiomes engage in both symbiotic and pathogenic interactions that have implications for human health. Symbiotic relationships involve mutual microbial exchange that contributes to immune system development and metabolic regulation in both hosts (Song et al., 2013). In contrast, pathogenic interactions may occur when wildlife act as reservoirs of emerging infectious agents, while domestic animals can function as amplifiers, facilitating zoonotic transmission to humans. Additionally, certain microorganisms are capable of causing similar diseases in both species. Collectively, these findings underscore the interconnectedness of human and animal health and suggest that the microbiome may serve as a critical link between human and veterinary medicine (Morand et al., 2014; Ma et al., 2023).

Although the number of microbial cells per gram is similar between the human gut and soil, soil harbors significantly greater species-level diversity. For instance, 1 gram of soil may contain approximately 4 × 10^3^ to 5 × 10^4^ microbial species, compared to about 4 × 10^2^ species in 1 gram of human feces (Blum et al., 2019). The soil microbiome plays a fundamental role in maintaining soil health, supporting plant productivity, and delivering critical ecosystem services (Vincze et al., 2024). High microbial diversity improves soil structure and fertility, root system architecture, and nutrient acquisition. It also contributes to plant nutrition, resilience to biotic and abiotic stress, and overall agricultural productivity. Moreover, it enhances the soil's adaptability to changing climate conditions, land-use patterns, and agronomic practices (Saleem et al., 2019).

According to the statement released in 2021 by the One Health High-Level Expert Panel, One Health is defined as an integrated, unifying approach that aims to sustainably balance and optimize the health of humans, animals, and ecosystems (World Health Organization, 2023). This approach emphasizes the close connections and interdependent relationships between human, animal, and environmental health, including ecosystems and plant life. As such, the One Health concept depends on coordinated collaboration across multiple sectors, disciplines, and communities to address complex challenges at the interface of these domains (Ma et al., 2023). Given that microorganisms are ubiquitous across humans, animals, and the environment, and substantial evidence exists for microbial strain sharing across these systems, the One Health approach is particularly relevant to microbiome research. Applying this approach enables a more comprehensive understanding of microbial transmission, co-evolution, and shared health outcomes across species and ecosystems (Ma et al., 2023; Tomasulo et al., 2024; Muhummed et al., 2025; Muloi et al., 2025).

One Health is a transdisciplinary framework that recognizes the interconnected health of humans, animals, plants, and the environment, aiming to improve overall well-being through a sustainable perspective (Ma et al., 2023). The Gulf Cooperation Council (GCC) represents a uniquely informative bioregion due to its distinct ecological and demographic characteristics, including arid environments, water scarcity, rapid urbanization, and food security challenges. Microbiome science is rapidly emerging as a transformative field with wide-ranging applications across multiple sectors. Recent advances in microbiome research and related technologies are enabling the integration of microbial data across disciplines, thereby enhancing its translational relevance for human, animal, and environmental health. As a result, there is a growing international movement to embed microbiome science within One Health strategies (Tomasulo et al., 2024). Collectively, these developments underscore the fundamental interconnectedness of biological systems and highlight the microbiome's pivotal role in advancing the One Health agenda, particularly in regions such as the GCC where environmental and demographic factors amplify its translational potential.

The GCC countries (Saudi Arabia, Qatar, Kuwait, United Arab Emirates, Oman, and Bahrain) are increasingly investing in health, agricultural, and environmental research as part of broader commitments to sustainable development (Zaidan et al., 2019). However, the region faces several interconnected One Health challenges, including high antimicrobial use and resistance, rapid urbanization, environmental constraints such as water scarcity and large-scale population movements associated with mass gatherings such as Hajj (Pao et al., 2025; Sheerah et al., 2025). These factors are likely to influence microbial ecosystems by altering microbial exposure, transmission dynamics, and selective pressures, which are key determinants of microbiome composition (The Integrative HMP (iHMP) Research Network Consortium, 2014). Despite the global surge in microbiome research, a comprehensive overview of the microbiome research landscape within the GCC countries remains lacking. To date, no systematic review has comprehensively examined how microbiome research in the GCC is distributed across human, animal, and environmental domains within a One Health framework. This systematic review aimed to systematically map and characterize microbiome research across human, animal, and environmental domains in the GCC within a One Health framework. It further aimed to identify critical knowledge gaps, research limitations, and areas of opportunity. By providing an evidence-based overview, this review aims to support future investment, drive innovation, and inform policy coordination, ultimately advancing the translation of microbiome science from discovery to application within the region.

## 2 Methods

### 2.1 Search terms and strategy

This systematic review was conducted in accordance with the Preferred Reporting Items for Systematic Reviews and Meta-Analyses (PRISMA) 2020 guidelines (Page et al., 2021). A comprehensive literature search was performed across the following electronic databases: MEDLINE via PubMed, ScienceDirect, Google Scholar, and EBSCO, covering microbiome-related studies published from database inception through January 31, 2025. No restrictions on study design, language, or publication status were applied during the initial search. A standardized search strategy was developed using combinations of the following keywords and Boolean operators: (microbiota OR microbiome OR microflora OR microbial community OR resistome OR phageome OR virome OR mycobiome OR metagenome OR metagenomics OR metabolome OR metabolomics OR metaproteome OR metaproteomics OR metatranscriptome OR metatranscriptomics) AND (human OR animal OR environmental OR marine OR sea OR air OR soil) AND (Gulf Cooperation Council OR GCC OR Saudi Arabia OR Qatar OR Kuwait OR United Arab Emirates OR Oman OR Bahrain). All retrieved records were exported and duplicates were removed prior to screening. Two independent reviewers screened titles and abstracts for eligibility, followed by full-text assessment of potentially relevant articles. Any discrepancies between reviewers were resolved through discussion and consensus. In addition to database searches, the reference lists of all included studies were manually screened to identify further eligible publications. To enhance search sensitivity and identify potentially relevant studies that may not have been captured through conventional database searches, the Connected Papers database^1^ was also used as a supplementary search tool to identify related articles based on citation mapping and semantic similarity.

### 2.2 Eligibility criteria

This review included original research articles published in English up to January 31, 2025. Studies were considered eligible if they were conducted in one or more GCC countries or if the analyzed samples originated from the GCC countries, including Saudi Arabia, Qatar, Kuwait, the United Arab Emirates, Oman, or Bahrain. Eligible human studies included participants who were citizens of the GCC countries, either within single-country investigations or multinational collaborations, regardless of age, sex, health status, study design, or healthcare setting. Animal studies were eligible if they involved animals residing within the GCC countries. Environmental studies were included if environmental samples (e.g., soil, water, air, or marine environments) were collected from locations within the GCC countries.

A broad range of study designs was considered, including observational studies (cross-sectional, case-control, and cohort studies), interventional studies (including randomized controlled trials), case reports and case series, and *in vitro* studies with microbiome-related outcomes relevant to GCC-derived samples. To be eligible, studies were required to report microbiome-related outcomes, including but not limited to microbial diversity, taxonomic composition, or functional profiling. No restrictions were imposed on microbiome investigation methods or analytical platforms. Therefore, studies employing sequencing-based approaches, PCR-based techniques, culture-dependent methods, metabolomics, or other microbiome-related analytical approaches were included. Studies were excluded if they were conducted entirely outside the GCC countries or did not report microbiome-related outcomes. This included studies that referenced the microbiome only conceptually or theoretically without employing any microbiome investigation methods. Excluded publication types comprised non-original research articles, including reviews, conference abstracts, study protocols, questionnaire-based studies, editorials, letters, and commentaries.

### 2.3 Study selection and data extraction

All records retrieved from the database searches were imported into Microsoft Excel (Microsoft Excel for Mac, Version 16.80), where duplicate entries were identified and removed. Study selection was conducted independently by two reviewers in two sequential stages according to the predefined eligibility criteria. In the first stage, titles and abstracts were screened to identify potentially relevant studies. In the second stage, the full texts of studies that met the initial screening criteria were assessed for final eligibility. Reviewer assessments were conducted independently and blinded to each other's decisions. Any disagreements were resolved through discussion, and when consensus could not be reached, a third reviewer was consulted.

Following study selection, two reviewers independently extracted data from all included studies using a structured data extraction form developed specifically for this review and aligned with its aim (Supplementary Tables 1–3). Data extraction was performed in Microsoft Excel and included the following variables: citation details, year of publication, country of study, study design, disease or research focus (for human microbiome studies), sample type, sample size, participant age and sex, and study period. Additional extracted variables included microbiome investigation methods and analytical platforms, study funding source, and a concise summary of key findings. For animal and environmental studies, the specific source and type of biological or environmental samples were also recorded.

Human sample types were categorized according to the Human Microbiome Project (HMP) body site classification framework (Turnbaugh et al., 2007; Peterson et al., 2009). Participant age was categorized into two groups: children (0–18 years) and adults (>18 years). Data extraction was completed independently by both reviewers, and the resulting datasets were compared to ensure consistency and accuracy. Any discrepancies were resolved through discussion and consensus. Following data consolidation, the finalized dataset was cross-checked by both reviewers to ensure completeness and data integrity prior to analysis. The study selection process was documented using a PRISMA 2020 flow diagram.

### 2.4 Risk of bias assessment

The methodological quality and risk of bias of the included studies were evaluated using established and study design-specific assessment tools. For observational human, animal, and environmental microbiome studies, reporting quality was assessed using the Strengthening the Reporting of Observational Studies in Epidemiology (STROBE) tool, which is designed for cohort, case-control, and cross-sectional study designs (von Elm et al., 2007). The STROBE tool comprises 22 items addressing methodological transparency, reporting of results, and completeness of study documentation. Each item was scored as “Yes” (1 point) if adequately reported or “No” (0 points) if inadequately reported, following previously published scoring approaches (Granate-Marques et al., 2019). Overall reporting quality scores were calculated for each study to enable comparative assessment across included observational studies. As the STROBE tool primarily evaluates reporting quality rather than methodological bias, it was used to assess transparency and completeness of reporting, which may influence the interpretability and reproducibility of findings (Supplementary Table 4).

The quality of interventional human microbiome studies was evaluated using the National Heart, Lung, and Blood Institute (NHLBI) Study Quality Assessment Tools^2^ This instrument includes 14 criteria addressing key methodological aspects such as randomization, allocation concealment, blinding, attrition, and other potential sources of bias (Supplementary Table 5A). Based on the overall assessment, studies were categorized as good, fair, or poor, reflecting the potential risk of bias associated with methodological limitations. For experimental animal microbiome studies, the Systematic Review Center for Laboratory Animal Experimentation (SYRCLE) risk of bias tool was applied (Hooijmans et al., 2014). This tool comprises 10 domains covering selection bias, performance bias, detection bias, attrition bias, and reporting bias. Each domain was rated as “Yes” (low risk of bias), “No” (high risk of bias), or “Unclear” when insufficient methodological detail was provided, in accordance with established SYRCLE guidance (Supplementary Table 5B; Hooijmans et al., 2014). The quality of environmental microbiome studies with experimental design was assessed using the Risk Of Bias In Non-randomized Studies of Interventions (ROBINS-I) tool (Sterne et al., 2016). This tool evaluates seven domains spanning pre-intervention, intervention, and post-intervention phases. Studies were classified as having low, moderate, serious, or critical risk of bias according to ROBINS-I criteria (Supplementary Table 5C; Sterne et al., 2016).

Different assessment tools were applied according to study design to ensure appropriate and domain-specific evaluation of methodological rigor and potential bias across human, animal, and environmental microbiome studies. All studies were independently assessed by two reviewers. Discrepancies in scoring or classification were resolved through discussion and consensus, and when necessary, a third reviewer was consulted.

### 2.5 Data synthesis and statistical analysis

Given the substantial heterogeneity across included studies in terms of study design, sample types, sequencing platforms, analytical pipelines, and reported outcomes, this review was conducted as a qualitative evidence synthesis rather than a quantitative meta-analysis or individual participant data meta-analysis. Quantitative pooling of effect estimates and reconstruction of raw datasets were not undertaken, as methodological differences across studies precluded meaningful statistical aggregation and could have resulted in misleading inferences (Page et al., 2021).

The primary aim of this review was to systematically map and characterize microbiome research conducted across the GCC countries within a One Health framework, rather than to quantify specific microbiome-disease associations. Accordingly, findings were synthesized descriptively to capture the scope, distribution, and methodological characteristics of human, animal, and environmental microbiome studies across the region. Extracted variables were summarized using frequencies and percentages, and results were organized according to study domain (human, animal, and environmental), country of origin, sample type, and microbiome analytical approaches. This approach enabled identification of research trends, methodological patterns, and critical knowledge gaps, thereby informing regional research priorities and opportunities for advancing integrated One Health microbiome research. Data management, descriptive analysis, and figure generation were performed using Microsoft Excel for Mac (version 16.80; Microsoft Corp., Redmond, WA, USA) and GraphPad Prism (version 10.6.1; GraphPad Software, San Diego, CA, USA). Conceptual illustrations and schematic figures were created using BioRender (BioRender.com).

## 3 Results

### 3.1 Literature search and study selection

**Figure 1:**
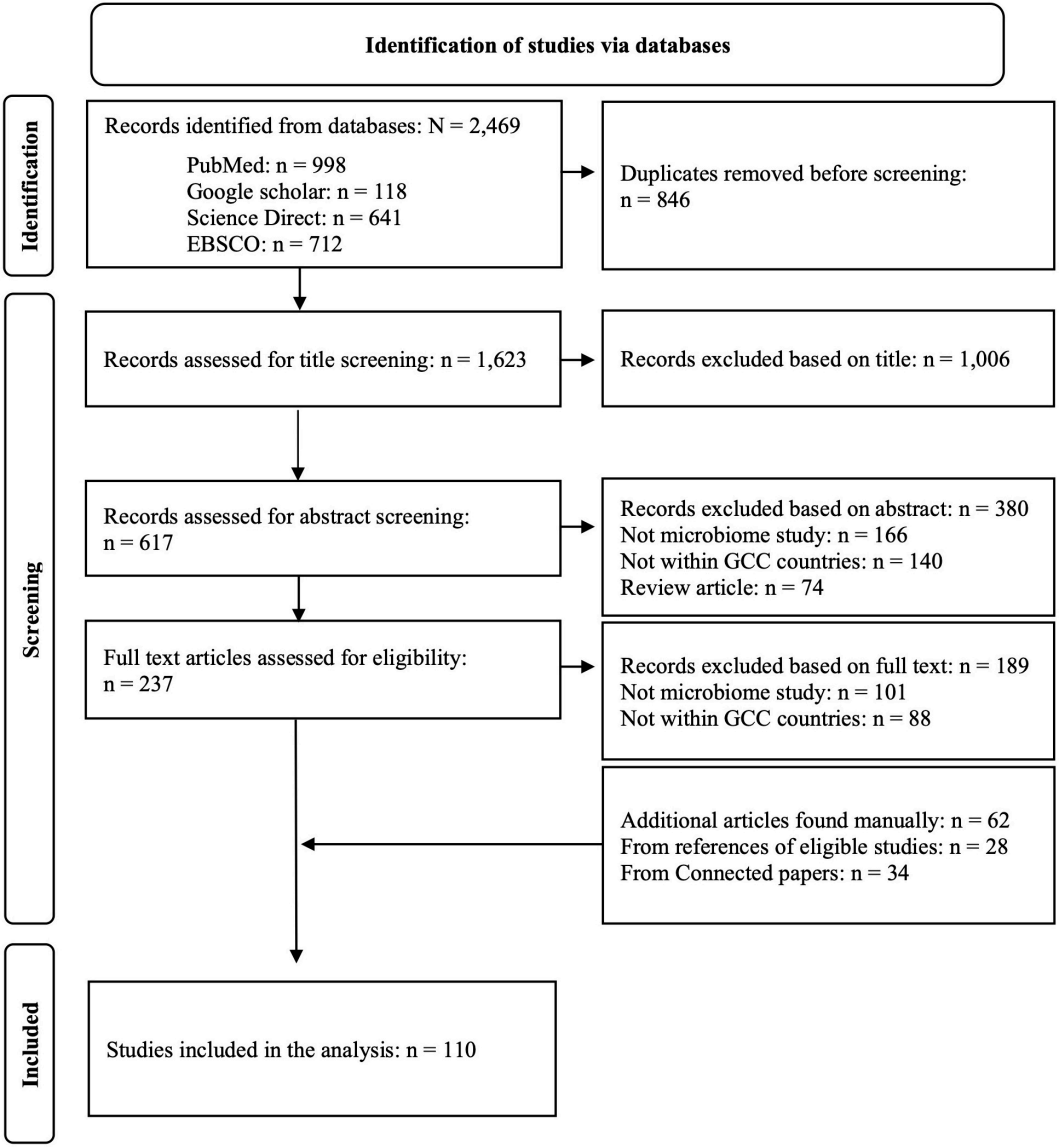
Preferred reporting items for systematic reviews and meta-analyses (PRISMA) 2020 flow diagram illustrating the study selection process for microbiome research conducted in the Gulf Cooperation Council (GCC) countries. The figure was generated using the PRISMA 2020 flow diagram template (accessed from http://www.prisma-statement.org/).

A comprehensive systematic literature search identified 2,469 records across four major electronic databases: PubMed, Google Scholar, ScienceDirect, and EBSCO. After removal of 846 duplicate records, 1,623 unique records remained and were screened by title, resulting in the exclusion of 1,006 studies. The remaining 617 records underwent abstract screening, during which 380 studies were excluded for not meeting the inclusion criteria, primarily because they were not microbiome-related studies, were not conducted within the GCC countries, or were review articles. A total of 237 full-text articles were subsequently assessed for eligibility. Of these, 189 were excluded following full-text review: 101 did not report microbiome-related outcomes and 88 were not conducted within the GCC countries or did not include samples originating from the GCC countries. In addition to database searches, 62 potentially relevant studies were identified through supplementary search approaches, including manual screening of reference lists (*n* = 28) and searches using the Connected Papers database (*n* = 34). In total, 110 studies met the predefined eligibility criteria and were included in the final systematic review. The study selection process is shown in the PRISMA 2020 flow diagram (Figure 1).

### 3.2 Risk of bias assessment

A total of 97 observational microbiome studies were included in the reporting quality assessment, comprising 52 observational human microbiome studies, nine observational animal microbiome studies, and 36 observational environmental microbiome studies.

The reporting quality of these studies was evaluated using the STROBE tool (Supplementary Table 6). The majority of included studies demonstrated high reporting quality. Only one observational human microbiome study (Alzahrani et al., 2021) was rated as having moderate reporting quality based on the STROBE scoring criteria, and no studies fell into the low-quality category.

The NHLBI quality assessment tool was applied to interventional human microbiome studies (*n* = 2; Supplementary Table 7A). Of these, one study was rated as fair quality (AlSarhan et al., 2021), while the other (Singh et al., 2020) was classified as poor quality, indicating methodological limitations and a higher potential risk of bias. The SYRCLE risk of bias tool was used to evaluate experimental animal microbiome studies (*n* = 3; Supplementary Table 7B). One study (Badger-Emeka et al., 2020) demonstrated an overall high risk of bias, with only the detection bias domain rated as low risk. The remaining two studies (Al-Asmakh et al., 2020; Al-Marzooqi et al., 2020) showed an overall lower risk of bias across most domains, although certain domains including selection, performance, and detection bias were rated as high risk due to insufficient methodological reporting. The ROBINS-I tool was applied to experimental environmental microbiome studies (*n* = 8; Supplementary Table 7C). Three studies were categorized as having a moderate risk of bias (Yaish et al., 2016; Bamigbade et al., 2024; Mousa et al., 2024b), four were classified as having a serious risk of bias (Baz et al., 2022; Al-Hadidi et al., 2024; Jayasree Subhash et al., 2024; Yaish et al., 2024), and one study was rated as having a critical risk of bias (Al-Sarawi et al., 2008), reflecting substantial methodological concerns.

Overall, the quality assessment indicated that most observational microbiome studies conducted in the GCC countries demonstrated high reporting quality. However, variability in methodological rigor was observed across experimental studies. These findings should be interpreted with caution due to the relatively small number of interventional and experimental studies included, which may limit the generalizability of the quality and risk-of-bias assessments, and underscores the need for improved adherence to standardized reporting practices and risk-of-bias mitigation strategies in future microbiome research across the region.

### 3.3 Overview of human, animal, and environmental microbiome research in GCC countries

**Figure 2:**
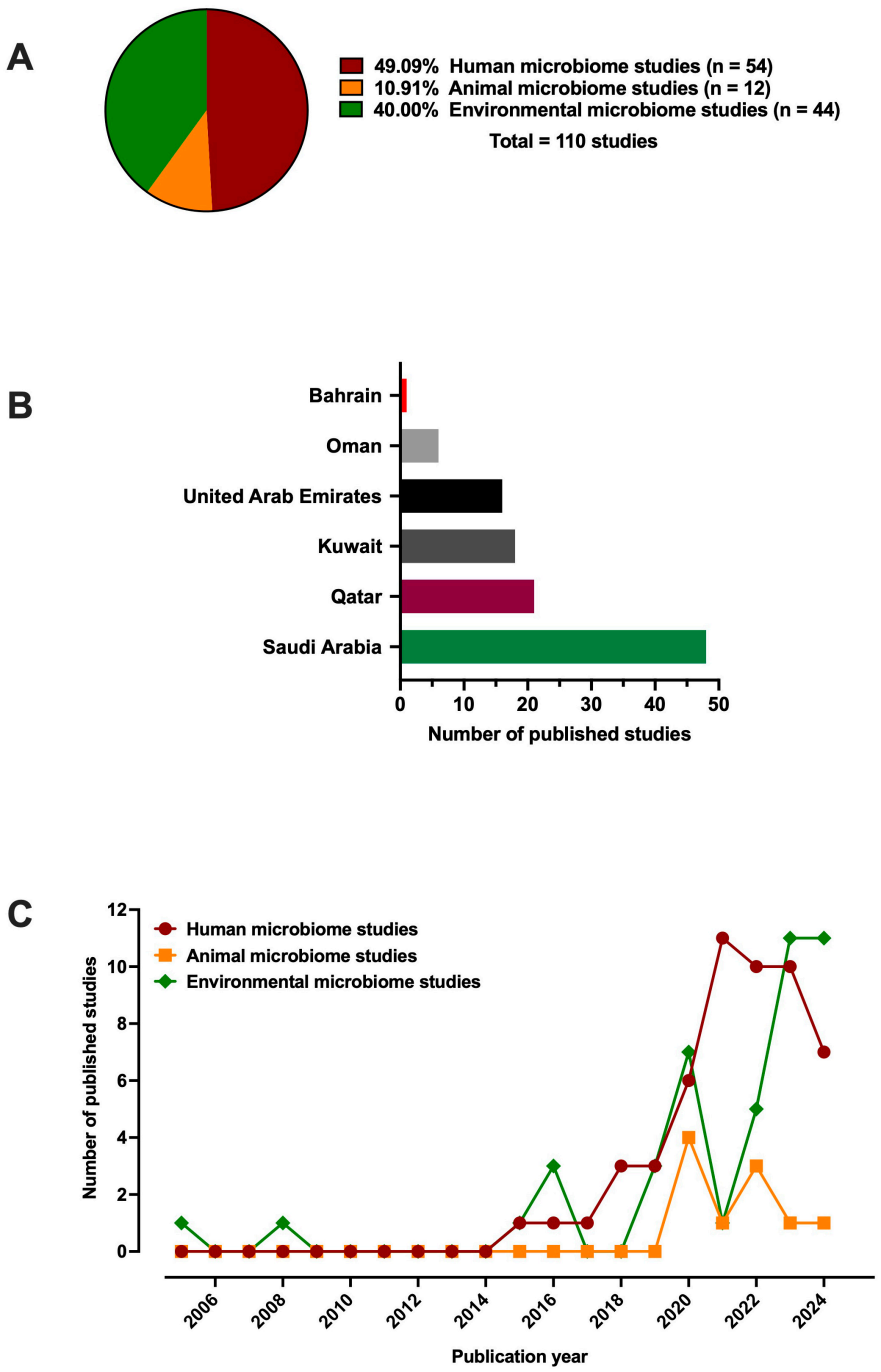
Overview of microbiome research in the Gulf Cooperation Council (GCC) countries. **(A)** Proportional distribution of all included studies, showing the relative contributions of human, animal, and environmental microbiome research. **(B)** Distribution of microbiome studies across the GCC countries based on the total number of included publications from each country. **(C)** Annual publication trends in microbiome research across the GCC countries from 2005 to 2024, stratified by study domain (human, animal, and environmental).

Microbiome research conducted in the GCC countries has predominantly focused on human studies, which represented nearly half of all included publications (49%; Figure 2A; Tables 1A, B). Environmental microbiome studies comprised the second-largest category (40%; Figure 2A; Tables 2A, B), whereas animal microbiome studies accounted for only 11% of the total (Figure 2A; Tables 3A, B). These findings indicate that microbiome research across the GCC countries has largely concentrated on human and environmental systems, with comparatively limited attention given to animal microbiomes. This imbalance suggests that current efforts remain largely sector-specific, with limited integration across human, animal, and environmental domains. The underrepresentation of animal microbiome studies may reflect gaps in veterinary research capacity, funding priorities, or limited integration with food security and agricultural systems, which are central to the One Health framework.

**Table 1A:** Data extracted from eligible human microbiome studies conducted in the Gulf Cooperation Council (GCC) countries and included in the current systematic review (*n* = 54).

Study no.	Citation and study title	Publication year	Country, sample origin	Study design	Disease focus	Sample type	Sample size and sex	Age group, years	Study period
**1**	Ahmad et al., 2025 Changes in energy homeostasis, gut peptides, and gut microbiota in Emiratis with obesity after bariatric surgery	2025	United Arab Emirates	Observation, cross section	Obesity	Stool	*N* = 19 *M, n* = 7 *F, n* = 12	Adults: 29.78 ± 2.29	October 2019–March 2021
**2**	Mustafa and Habibi, 2024 Spatial variations in the nasal microbiota of staff working in a healthcare-associated research core facility	2024	Kuwait	Observation, cross section	No specific disease was mentioned	Anterior nares	*N* = 10 (authors did not clarify sex)	Adults: laboratory staff, no specific age was mentioned	No mention
**3**	Alahdal et al., 2024 Gut microbiota composition in patients with Crohn's disease in Saudi Arabia	2024	Saudi Arabia	Observation, case control	Crohn's disease	Stool	*N* = 80 *M, n* = 49 *F, n* = 31	Adults: ≥17	No mention
**4**	Benslimane et al., 2024 Metabarcoding analysis of oral microbiome during pregnancy	2024	Qatar	Observation, cross section	Pregnancy	Saliva	*N* = 5 (five Qatari females)	Adults: 28.86 ± 5.12	No mention
**5**	Murugesan et al., 2024 Microbial and proteomic signatures of type 2 diabetes in an Arab population	2024	Qatar	Observation, case control	Type 2 diabetes mellitus	Saliva	*N* = 2,974 *M, n* = 1,430 *F, n* = 1,544	Adults: >17	No mention
**6**	Abraham et al., 2024 Molecular analyses indicate profuse bacterial diversity in primary and post-treatment endodontic infections within a cohort from the United Arab Emirates-a preliminary study	2024	United Arab Emirates	Observation, cross section	Endodontic infections	Infected intracanal samples	*N* = 20 (authors did not clarify sex)	Adults: ≥18	January–June 2019
**7**	Lu et al., 2024 Sputum production and salivary microbiome in COVID-19 patients reveals oral-lung axis	2024	Kuwait	Observation, cross section	COVID-19	Saliva and blood	*N* = 50 *M, n* = 28 *F, n* = 22	Adults: mean age was 47.3	July–September 2020
**8**	Augustine et al., 2024 Immunoglobulin-coating patterns reveal altered humoral responses to gut bacteria in pediatric cow milk allergies	2024	Qatar	Observation, case control	Allergy	Stool	*N* = 38 (all were males)	Children: 1–4	No mention
**9**	Alqedari et al., 2024 Host-microbiome associations in saliva predict COVID-19 severity	2024	Kuwait	Observation, case control	COVID-19	Saliva	*N* = 80 *M, n* = 44 *F, n* = 36	Adults: Less than 64% of participants were >50 years	July–September 2020
**10**	Yasir et al., 2023b Analysis of the nasopharyngeal microbiome and respiratory pathogens in COVID-19 patients from Saudi Arabia	2023	Saudi Arabia	Observation, case control	COVID-19	Nasopharyngeal swabs	*N* = 107 (authors did not clarify sex)	Adults: COVID-19 patients: 44.2 ± 17.6 Control group: 38.4 ± 8.0	February–June 2022
**11**	Ahmad et al., 2023 Association of the gut microbiota with clinical variables in obese and lean Emirati subjects	2023	United Arab Emirates	Observation, cross section	Obesity	Stool	*N* = 74 *M, n* = 28 *F, n* = 46	Adults: 18–60	October 2019–March 2021
**12**	Al-Amrah et al., 2023 Composition of the gut microbiota in patients with inflammatory bowel disease in Saudi Arabia: a pilot study	2023	Saudi Arabia	Observation, case control	Inflammatory bowel disease	Stool	*N* = 21 *M, n* = 8 *F, n* = 13	Adults: Case: 28.91 ± 6.4 Control: 32.4 ± 5.9	No mention
**13**	Alhhazmi et al., 2023 Identification of gut microbiota profile associated with colorectal cancer in Saudi population	2023	Saudi Arabia	Observation, case control	Colorectal cancer	Stool	*N* = 50 *M, n* = 36 *F, n* = 14	Adults: Case: 54.3 ± 14.2 Control: 47.4 ± 11.7	No mention
**14**	Alsulaiman et al., 2023 Gut microbiota analyses of inflammatory bowel diseases from a representative Saudi population	2023	Saudi Arabia	Observation, case control	Inflammatory bowel disease	Stool	*N* = 343 *M, n* = 219 *F, n* = 124	Adults: 57.5 ± 12.5	2015–2019
**15**	Alyousef et al., 2023 Oral microbiota analyses of pediatric Saudi population reveals signatures of dental caries	2023	Saudi Arabia	Observation, cross section	Dental caries	Saliva	*N* = 400 (authors did not clarify sex)	Children: 6–12	2019–2020
**16**	Murugesan and Al Khodor, 2023 Salivary microbiome and hypertension in the Qatari population	2023	Qatar	Observation, case control	Hypertension	Saliva	*N* = 1,190 *M, n* = 725 *F, n* = 465	Adults: mean age was 43	No mention
**17**	Al-Khaldy et al., 2023 Serum vitamin D level and gut microbiota in women	2023	Saudi Arabia	Observation, case control	Obesity and vitamin D deficiency	Stool and blood	*N* = 92 (all were females)	Adults: 18–25	January 2019–March 2020
**18**	Aljuraiban et al., 2023 Types of fiber and gut microbiota composition and diversity among Arab females	2023	Saudi Arabia	Observation, cross section	Obesity	Stool	*N* = 92 (all were females)	Adults: ≥18	January 2019–March 2020
**19**	Aljazairy et al., 2022 Influence of adiposity on the gut microbiota composition of Arab women: a case-control study	2022	Saudi Arabia	Observation, case control	Obesity	Stool	*N* = 92 (all were females)	Adults: 18–25	January 2019–March 2020
**20**	Al-Musharaf et al., 2022 Vitamin B12 status and gut microbiota among Saudi females with obesity	2022	Saudi Arabia	Observation, case control	Obesity	Stool	*N* = 92 (all were females)	Adults: 19–25	No mention
**21**	Hijazi et al., 2022 Viral metagenomics analysis of stool specimens from children with unresolved gastroenteritis in Qatar	2022	Qatar	Observation, cross section	Acute gastroenteritis	Stool	*N* = 29 *M, n* = 17 *F, n* = 12	Children: < 5	June 2016–August 2019
**22**	Wehedy et al., 2022 Characterization of the urinary metagenome and virome in healthy children	2022	Qatar	Observation, cross section	Healthy	Urine	*N* = 40 *M, n* = 25 *F, n* = 15	Children: 1–17	September 2020–December 2021
**23**	Al Shareef et al., 2022 Diversity in microbiota between Indian and Emiratis ethnicities is associated with benign prostatic hyperplasia	2022	United Arab Emirates	Observation, cross section	Benign prostatic hyperplasia	Prostate tissue	*N* = 3 (three Emiratis males)	Adults: 58–66	No mention
**24**	Al-Muhanna et al., 2022 Gut microbiota analyses of Saudi populations for type 2 diabetes-related phenotypes reveals significant association	2022	Saudi Arabia	Observation, case control	Type 2 diabetes	Stool	*N* = 580 *M, n* = 316 *F, n* = 264	Adults: 30–75	2015–2019
**25**	El Mouzan et al., 2022a Intestinal microbiota profile in healthy Saudi children: the bacterial domain	2022	Saudi Arabia	Observation, cross section	Healthy	Stool	*N* = 20 *M, n* = 7 *F, n* = 13	Children: 6.8–15.4	No mention
**26**	El Mouzan et al., 2022b Microbiota profile of new onset celiac disease in children in Saudi Arabia	2022	Saudi Arabia	Observation, case control	Celiac disease	Mucosal samples from the duodenum and stool	*N* = 79 *M, n* = 26 *F, n* = 53	Children: < 18	No mention
**27**	Al-Marzooq et al., 2022 Supragingival microbiome alternations as a consequence of smoking different tobacco types and its relation to dental caries	2022	United Arab Emirates	Observation, cross section	Dental caries	Supragingival plaque	*N* = 40 *M, n* = 34 *F, n* = 6	Adults: 18–60	October 2018–October 2019
**28**	Singh et al., 2022 Tipping the balance: vitamin D inadequacy in children impacts the major gut bacterial phyla	2022	Qatar	Observation, cross section	Healthy	Stool and blood	*N* = 112 (authors did not clarify sex)	Children: 4–14	No mention
**29**	Alzahrani et al., 2021 Direct DNA sequencing-based analysis of microbiota associated with hematological malignancies in the eastern province of Saudi Arabia	2021	Saudi Arabia	Observation, case control	Bloodstream infections	Blood	*N* = 100 (authors did not clarify sex)	Adults: 18–74	March 2017–February 2019
**30**	Lakshmanan et al., 2021 *Akkermansia*, a possible microbial marker for poor glycemic control in Qataris children consuming Arabic diet—a pilot study on pediatric T1DM in Qatar	2021	Qatar	Observation, cross section	Type 1 diabetes mellitus	Stool	*N* = 14 (authors did not clarify sex)	Children: 6–12	No mention
**31**	Murugesan et al., 2021 Can the salivary microbiome predict cardiovascular diseases? lessons learned from the Qatari population	2021	Qatar	Observation, cross section	Cardiovascular disease	Saliva	*N* = 2,974 *M, n* = 1,432 *F, n* = 1,542	Adults: 35–55	No mention
**32**	Habibi et al., 2021 Composition of nasal bacterial community and its seasonal variation in health care workers stationed in a clinical research laboratory	2021	Kuwait	Observation, cross section	No specific disease was mentioned	Nasal swab	*N* = 17 (authors did not clarify sex)	Adults: 18–60	No mention
**33**	Singh et al., 2021 Distinctive microbial signatures and gut-brain crosstalk in pediatric patients with coeliac disease and type 1 diabetes mellitus	2021	Qatar	Observation, cross section	Type 1 diabetes mellitus and celiac disease	Stool	*N* = 58 (authors did not clarify sex)	Children: 8–18	No mention
**34**	Al Bataineh et al., 2021 Gut Microbiota interplay with COVID-19 reveals links to host lipid metabolism among Middle Eastern populations	2021	United Arab Emirates	Observation, case control	COVID-19	Stool	*N* = 143 *M, n* = 66 *F, n* = 77	Adults: ≥18	July–August 2020
**35**	Sohail et al., 2021 Microbiome profiling of Rotavirus infected children suffering from acute gastroenteritis	2021	Qatar	Observation, case control	Acute gastroenteritis	Stool	*N* = 47 (authors did not clarify sex)	Children: 20–13.5 months	No mention
**36**	Alqattan et al., 2021 Microbiome signature and diversity profiling of normal skin of human in Saudi Arabia	2021	Saudi Arabia	Observation, cross section	Healthy	Skin swab	*N* = 8 *M, n* = 4 *F, n* = 4	Adults: 20–37	No mention
**37**	Alqaderi et al., 2021 Salivary microbiome diversity in Kuwaiti adolescents with varied body mass index—a pilot study	2021	Kuwait	Observation, cross section	Overweight and obesity	Saliva	*N* = 23 (authors did not clarify sex)	Adolescents (authors did not specify age)	No mention
**38**	AlSarhan et al., 2021 Short-term improvement of clinical parameters and microbial diversity in periodontitis patients following Indocyanine green-based antimicrobial photodynamic therapy: a randomized single-blind split-mouth cohort	2021	Saudi Arabia	Intervention, single-blind, split-mouth cohort	Periodontitis	Subgingival plaque	*N* = 20 (authors did not clarify sex)	Adults: >30	January 2018–December 2018
**39**	Abdulhaq et al., 2021 Tongue microbiome in children with autism spectrum disorder	2021	Saudi Arabia	Observation, case control	Autism spectrum disorder	Tongue scraping	*N* = 63 *M, n* = 34 *F, n* = 29	Children: mean age was 9.24–10.03	2018–2019
**40**	Plummer et al., 2020 Gut microbiome of native Arab Kuwaitis	2020	Kuwait	Observation, cross section	Healthy	Stool	*N* = 25 *M, n* = 10 *F, n* = 15	Adults: 24–57	No mention
**41**	Masoodi et al., 2020 Microbial dysbiosis in irritable bowel syndrome: a single-center metagenomic study in Saudi Arabia	2020	Saudi Arabia	Observation, case control	Irritable bowel syndrome	Gastrointestinal, mucosal wash	*N* = 100 (authors did not clarify sex)	Adults: 19–65	July 2010–November 2012
**42**	Moubareck et al., 2020 Profiles of human milk oligosaccharides and their relations to the milk microbiota of breastfeeding mothers in Dubai	2020	United Arab Emirates	Observation, cross section	Healthy	Breast milk	*N* = 30 (16 were Emirati females)	Adults: >17	March–April 2018
**43**	Murugesan et al., 2020 Profiling the salivary microbiome of the Qatari population	2020	Qatar	Observation, cross section	Healthy	Saliva	*N* = 997 *M, n* = 442 *F, n* = 555	Adults: 38.2 ± 12.0	No mention
**44**	Al Bataineh et al., 2020 Revealing links between gut microbiome and its fungal community in type 2 diabetes mellitus among Emirati subjects: a pilot study	2020	United Arab Emirates	Observation, case control	Type 2 diabetes mellitus	Stool	*N* = 50 *M, n* = 12 *F, n* = 38	Adults: 21–67	No mention
**45**	Singh et al., 2020 The potential role of vitamin D supplementation as a gut microbiota modifier in healthy individuals	2020	Qatar	Intervention	Vitamin D deficiency	Stool and blood	*N* = 80 (all were females)	Adults: 18–30	March–September 2018
**46**	Khan et al., 2019 Evaluation of gut bacterial community composition and antimicrobial resistome in pregnant and non-pregnant women from Saudi population	2019	Saudi Arabia	Observation, case control	Pregnancy	Stool	*N* = 24 (all were females)	Adults: < 50	No mention
**47**	Sohail et al., 2019 Profiling the oral microbiome and plasma biochemistry of obese hyperglycemic subjects in Qatar	2019	Qatar	Observation, case control	Obesity with hyperglycemia (pre-diabetes)	Saliva	*N* = 73 (all were males)	Adults: ≥30	No mention
**48**	Shami et al., 2019 The prevalence of the culturable human skin aerobic bacteria in Riyadh, Saudi Arabia	2019	Saudi Arabia	Observation, cross section	Healthy	Skin swab	*N* = 80 (authors did not clarify sex)	Adults: mean age was 62, 51, 20, and 21	No mention
**49**	Madi et al., 2018 Metagenomic analysis of viral diversity in respiratory samples from patients with respiratory tract infections in Kuwait	2018	Kuwait	Observation, cross section	Respiratory tract infections	Nasopharyngeal aspirates/wash, nasopharyngeal swab, bronchoalveolar lavage, tracheal aspirates, sputum, throat swabs, and nasal swabs	*N* = 86 *M, n* = 30 *F, n* = 56	Infants, children, and adults: 3 days−89	2015–2016
**50**	Alomair et al., 2018 Colonic mucosal microbiota in colorectal cancer: a single-center metagenomic study in Saudi Arabia	2018	Saudi Arabia	Observation, case control	Colorectal cancer	Colonic mucosa	*N* = 58 (authors did not clarify sex)	Adults: 38–77	July 2010–November 2012
**51**	Vallès et al., 2018 Types of tobacco consumption and the oral microbiome in the United Arab Emirates Healthy Future (UAEHFS) Pilot Study	2018	United Arab Emirates	Observation, cross section	Healthy	Mouthwash	*N* = 330 *M, n* = 226 *F, n* = 104	Adults: ≥18	December 2014–April 2015
**52**	Al-Rezaki et al., 2017 Assessment of the microbiome collected from the reproductive tracts of women from Saudi Arabia and its potential influence on infertility	2017	Saudi Arabia	Observation, case control	Infertility	Cervicovaginal fluid and vaginal swabs	*N* = 36 (all were females)	Adults: females at reproductive age	No mention
**53**	Angelakis et al., 2016 Gut microbiome and dietary patterns in different Saudi populations and monkeys	2016	Saudi Arabia	Observation, cross section	Healthy	Stool	*N* = 28 *M, n* = 26 *F, n* = 2	Adults: >17	No mention
**54**	Yasir et al., 2015a Comparison of the gut microbiota of people in France and Saudi Arabia	2015	Saudi Arabia	Observation, cross section	Obesity	Stool	*N* = 18 (all were males)	Adults: >17	No mention

M, Male; F, Female.

**Table 1B:** Data extracted from eligible human microbiome studies conducted in the Gulf Cooperation Council (GCC) countries and included in the current systematic review (*n* = 54).

Study no.	Citation and study title	Method	Platform	Sponsor	Summary of findings
**1**	Ahmad et al., 2025 Changes in energy homeostasis, gut peptides, and gut microbiota in Emiratis with obesity after bariatric surgery	16S rRNA gene sequencing	Illumina MiSeq	• Zayed University, United Arab Emirates • Dutch Province of Limburg with a grant to the Center for Healthy Eating and Food Innovation of Maastricht University, Netherlands	Changes in gut peptides, hormones, and microbiota may partially explain the weight-loss effects of bariatric surgery.
**2**	Mustafa and Habibi, 2024 Spatial variations in the nasal microbiota of staff working in a healthcare-associated research core facility	16S rRNA gene sequencing	Illumina MiSeq	• Kuwait University, Kuwait	Findings suggest that healthcare support staff may be exposed to occupational microbial shifts, where slight changes in bacterial composition could have adverse effects, suggesting the need for ongoing monitoring.
**3**	Alahdal et al., 2024 Gut microbiota composition in patients with Crohn's disease in Saudi Arabia	16S rRNA gene sequencing	Illumina (authors did not mention which sequencing platform)	• Princess Nourah bint Abdulrahman University, Saudi Arabia	Alterations in *Firmicutes, Proteobacteria, Bacteroidetes*, and increased *Fusobacteria* levels were associated with Crohn's disease activity and severity. Smoking, older age, and male sex were linked to more severe disease and reduced microbial diversity.
**4**	Benslimane et al., 2024 Metabarcoding analysis of oral microbiome during pregnancy	16S rRNA gene sequencing	Oxford Nanopore Technology (authors did not mention which sequencing platform)	• Qatar National Research Fund, Qatar • Qatar University, Qatar	Significant changes in clinical and microbial parameters during pregnancy highlight the complex relationship between maternal health and the microbiome.
**5**	Murugesan et al., 2024 Microbial and proteomic signatures of type 2 diabetes in an Arab population	16S rRNA gene sequencing	Illumina MiSeq	• Qatar National Research Fund, Qatar • Qatar genome program, Qatar	Salivary microbiota composition differed significantly between pre-diabetic and diabetic individuals compared to non-diabetic controls.
**6**	Abraham et al., 2024 Molecular analyses indicate profuse bacterial diversity in primary and post- treatment endodontic infections within a cohort from the United Arab Emirates-a preliminary study	16S rRNA gene sequencing	Ion S5 XL semiconductor sequencer using Ion 520 chip	• University of Sharjah, United Arab Emirates	Significant differences in microbiome composition and diversity were observed between primary and post-treatment endodontic infections in a United Arab Emirates cohort.
**7**	Lu et al., 2024 Sputum production and salivary microbiome in COVID-19 patients reveals oral-lung axis	16S rRNA gene sequencing	Illumina MiSeq	• J. Craig Venter Institute, CA, USA • Kuwait Ministry of Health, Kuwait • Dasman Diabetes Institute, Kuwait • L'Oréal UNESCO • National Institutes of Health (NIH)	Differences in oral bacterial composition and inflammatory cytokine levels were observed between patients with and without sputum production, indicating distinct host immune responses.
**8**	Augustine et al., 2024 Immunoglobulin-coating patterns reveal altered humoral responses to gut bacteria in pediatric cow milk allergies	16S rRNA gene sequencing	Illumina MiSeq	• Sidra medicine internal research fund, Qatar	Pathogenic bacteria uniquely associated with different allergic disease types may contribute to disease-specific pathological phenotypes.
**9**	Alqedari et al., 2024 Host-microbiome associations in saliva predict COVID-19 severity	16S rRNA gene sequencing	Illumina MiSeq	• Kuwait Ministry of Health, Kuwait • Dasman Diabetes Institute, Kuwait • L'Oréal-UNESCO • National Institute of Health • J. Craig Venter Institute, CA, USA	Oral microbiome profiles and salivary cytokines were associated with COVID-19 status and severity, suggesting their potential as predictive markers and offering insights into disease pathogenesis in immunologically naïve individuals.
**10**	Yasir et al., 2023b Analysis of the nasopharyngeal microbiome and respiratory pathogens in COVID-19 patients from Saudi Arabia	16S rRNA gene sequencing	Illumina MiSeq	• Jameel Fund for Infectious Disease Research and Innovation, Saudi Arabia	While overall nasopharyngeal bacterial composition remained similar, specific taxa differed between healthy controls and COVID-19 patients. COVID-19 cases showed increased respiratory pathogens, including probable *K. pneumoniae* colonization.
**11**	Ahmad et al., 2023 Association of the gut microbiota with clinical variables in obese and lean Emirati subjects	16S rRNA gene sequencing	Illumina MiSeq	• Zayed University, United Arab Emirates • Center for Healthy Eating and Food Innovation of Maastricht University, Netherlands	Significant differences in gut microbiota were observed between obese and lean adult Emiratis, with specific microbial genera associated with obesity.
**12**	Al-Amrah et al., 2023 Composition of the gut microbiota in patients with inflammatory bowel disease in Saudi Arabia: A pilot study	16S rRNA gene sequencing	Illumina MiSeq	• None	The study revealed differences in microbiota diversity between Saudi patients with inflammatory bowel disease and healthy controls.
**13**	Alhhazmi et al., 2023 Identification of gut microbiota profile associated with colorectal cancer in Saudi population	16S rRNA gene sequencing	Illumina (authors did not mention which sequencing platform)	• Ministry of Education, Saudi Arabia	Findings suggest that dysbiosis and increased microbial functional activity may contribute to the progression of advanced colorectal cancer.
**14**	Alsulaiman et al., 2023 Gut microbiota analyses of inflammatory bowel diseases from a representative Saudi population	16S rRNA gene sequencing	Illumina NovaSeq 6000	• King Abdulaziz City for Science and Technology, Saudi Arabia	Significant differences in gut microbiota composition were observed among Saudi patients with ulcerative colitis, Crohn's disease, and healthy controls.
**15**	Alyousef et al., 2023 Oral microbiota analyses of pediatric Saudi population reveals signatures of dental caries	16S rRNA gene sequencing	Illumina NextSeq 2000	• King Abdulaziz City for Science and Technology, Saudi Arabia	Oral microbiota analysis in a representative Saudi population revealed distinct abundance and diversity patterns associated with high and low dental caries levels.
**16**	Murugesan and Al Khodor, 2023 Salivary microbiome and hypertension in the Qatari population	16S rRNA gene sequencing	Illumina MiSeq	• Qatar National Research Fund, Qatar • Qatar genome program, Qatar	Salivary microbiome composition varied across blood pressure categories. Protective genera (e.g., *Haemophilus, Prevotella*, and *Neisseria*) were enriched in normotensive individuals, while *Bacteroides* and *Lactobacillus* were more abundant in hypertensive groups, potentially influencing blood pressure regulation via metabolic pathways.
**17**	Al-Khaldy et al., 2023 Serum vitamin D level and gut microbiota in women	Shotgun metagenomic sequencing	Illumina HiSeq 4000	• Deputyship for Research and Innovation, Ministry of Education, Saudi Arabia	Findings suggest that vitamin D may influence gut microbiota composition by suppressing pathogenic bacteria and supporting beneficial strains.
**18**	Aljuraiban et al., 2023 Types of fiber and gut microbiota composition and diversity among Arab females	Shotgun metagenomic sequencing	Illumina (authors did not mention which sequencing platform)	• Deputyship for Research and Innovation, Ministry of Education, Saudi Arabia	Insoluble dietary fiber intake was positively associated with gut microbiota health.
**19**	Aljazairy et al., 2022 Influence of adiposity on the gut microbiota composition of Arab women: a case-control study	Shotgun metagenomic sequencing	Illumina (authors did not mention which sequencing platform)	• Deputyship for Research and Innovation, Ministry of Education, Saudi Arabia	The study found an association between gut microbiota composition and obesity in Saudi women, linked to specific obesity-related markers.
**20**	Al-Musharaf et al., 2022 Vitamin B12 status and gut microbiota among Saudi females with obesity	Shotgun metagenomic sequencing	Illumina (authors did not mention which sequencing platform)	• Deputyship for Research and Innovation, Ministry of Education, Saudi Arabia	Gut microbiota abundance and diversity were associated with vitamin B12 levels and obesity in young females.
**21**	Hijazi et al., 2022 Viral metagenomics analysis of stool specimens from children with unresolved gastroenteritis in Qatar	Shotgun metagenomic sequencing	Illumina HiSeq	• Hamad Medical Corporation, Qatar • Qatar National Research Fund, Qatar	Viral metagenomics proved effective in identifying the cause of acute gastroenteritis when standard molecular diagnostic assays were inconclusive.
**22**	Wehedy et al., 2022 Characterization of the urinary metagenome and virome in healthy children	Shotgun metagenomic sequencing	Illumina MiSeq	• Sidra medicine internal research fund, Qatar	Optimized protocols enabled sex-specific identification of urinary bacteriome, mycobiome, and virome profiles in healthy children using 3–10 mL urine samples.
**23**	Al Shareef et al., 2022 Diversity in microbiota between Indian and Emiratis ethnicities is associated with benign prostatic hyperplasia	16S rRNA gene sequencing	Illumina (authors did not mention which sequencing platform)	• University of Sharjah, United Arab Emirates	Differences in bacterial community structure were observed between Indian and Emirati participants.
**24**	Al-Muhanna et al., 2022 Gut microbiota analyses of Saudi populations for type 2 diabetes-related phenotypes reveals significant association	16S rRNA gene sequencing	Illumina MiSeq	• King Abdulaziz City for Science and Technology, Saudi Arabia	Gut microbiota composition differed between Saudi individuals with and without type 2 diabetes, showing distinct diversity patterns compared to Western populations.
**25**	El Mouzan et al., 2022a Intestinal microbiota profile in healthy Saudi children: the bacterial domain	16S rRNA gene sequencing	Illumina HiSeq	• King Saud University, Saudi Arabia	Healthy Saudi children exhibited a distinct intestinal microbiome profile compared to other populations.
**26**	El Mouzan et al., 2022b Microbiota profile of new onset celiac disease in children in Saudi Arabia	Shotgun metagenomic sequencing	Illumina HiSeq	• King Saud University, Saudi Arabia	Bacterial profiles from mucosal and fecal samples of newly diagnosed children on a gluten-containing diet showed a strong association with celiac disease.
**27**	Al-Marzooq et al., 2022 Supragingival microbiome alternations as a consequence of smoking different tobacco types and its relation to dental caries	16S rRNA gene sequencing	Oxford Nanopore Technology, MinION	• Al Jalila Foundation for Medical Research, United Arab Emirates	Tobacco use, including cigarettes, medwakh, and shisha, significantly altered the supragingival plaque microbiome, with distinct effects observed between individuals with varying levels of dental caries.
**28**	Singh et al., 2022 Tipping the balance: vitamin D inadequacy in children impacts the major gut bacterial phyla	16S rRNA gene sequencing	Illumina MiSeq	• Sidra medicine internal research fund, Qatar	Pediatric vitamin D deficiency was associated with notable alterations in gut microbiota composition.
**29**	Alzahrani et al., 2021 Direct DNA sequencing-based analysis of microbiota associated with hematological malignancies in the eastern province of Saudi Arabia	16S rRNA gene sequencing	Illumina MiSeq	• Imam Abdulrahman bin Faisal University, Saudi Arabia	Polymicrobial bloodstream infections were common in patients with hematological malignancies. Next-generation sequencing enabled rapid and specific pathogen identification, supporting its potential use in routine infection monitoring.
**30**	Lakshmanan et al., 2021 *Akkermansia*, a possible microbial marker for poor glycemic control in Qataris children consuming Arabic diet—a pilot study on pediatric T1DM in Qatar	16S rRNA gene sequencing	Illumina MiSeq	• Sidra medicine internal research fund, Qatar	In poorly controlled Qatari children with type 1 diabetes, *Akkermansia* abundance was influenced by the Arabic diet, suggesting potential for personalized dietary interventions.
**31**	Murugesan et al., 2021 Can the salivary microbiome predict cardiovascular diseases? lessons learned from the Qatari population	16S rRNA gene sequencing	Illumina MiSeq	• Qatar National Research Fund, Qatar • Qatar Genome Program, Qatar	Salivary microbiome composition differed significantly between individuals at high and low risk for cardiovascular disease.
**32**	Habibi et al., 2021 Composition of nasal bacterial community and its seasonal variation in health care workers stationed in a clinical research laboratory	16S rRNA gene sequencing	Illumina MiSeq	• Kuwait University, Kuwait	Seasonal variation in indoor and outdoor environments influenced nasal bacterial community composition among healthcare professionals.
**33**	Singh et al., 2021 Distinctive microbial signatures and gut-brain crosstalk in pediatric patients with coeliac disease and type 1 diabetes mellitus	16S rRNA gene sequencing	Illumina MiSeq	• Sidra medicine internal research fund, Qatar	Distinct gut microbial features at the amplicon sequence variant level differentiated children with celiac disease from those with type 1 diabetes, with specific signatures also linked to small fiber neuropathy.
**34**	Al Bataineh et al., 2021 Gut Microbiota interplay with COVID-19 reveals links to host lipid metabolism among Middle Eastern populations	16S rRNA gene sequencing	Illumina MiSeq	• Khalifa and Sharjah Universities, United Arab Emirates	The study suggests a potential protective role of the gut microbiota against SARS-CoV-2 infection.
**35**	Sohail et al., 2021 Microbiome profiling of Rotavirus infected children suffering from acute gastroenteritis	16S rRNA gene sequencing	Illumina MiSeq	• Qatar National Research Fund, Qatar • Qatar University, Qatar	Distinct, dose-dependent microbial signatures were associated with rotavirus vaccine response, suggesting that modulating the microbiota could enhance vaccine efficacy.
**36**	Alqattan et al., 2021 Microbiome signature and diversity profiling of normal skin of human in Saudi Arabia	16S rRNA gene sequencing	Illumina (authors did not mention which sequencing platform)	• None	The study identified skin microbiome signatures specific to individuals in Saudi Arabia, providing a foundation for future biomarker research in skin diseases such as atopic dermatitis, psoriasis, and acne vulgaris.
**37**	Alqaderi et al., 2021 Salivary microbiome diversity in Kuwaiti adolescents with varied body mass index—a pilot study	16S rRNA gene sequencing	Illumina MiSeq	• Dasman Diabetes Institute, Kuwait	The pilot study suggested that altered salivary microbiome composition in overweight and obese individuals may indicate increased susceptibility to oral diseases.
**38**	AlSarhan et al., 2021 Short-term improvement of clinical parameters and microbial diversity in periodontitis patients following Indocyanine green-based antimicrobial photodynamic therapy: a randomized single-blind split-mouth cohort	16S rRNA gene sequencing	Illumina MiSeq	• King Saud University, Saudi Arabia	Antimicrobial photodynamic therapy, used alongside scaling and root planning, significantly improved periodontal clinical parameters in chronic periodontitis patients.
**39**	Abdulhaq et al., 2021 Tongue microbiome in children with autism spectrum disorder	16S rRNA gene sequencing	Illumina MiSeq	• Jazan University, Saudi Arabia	No significant differences were observed in the tongue microbiome between children with autism spectrum disorder and healthy controls.
**40**	Plummer et al., 2020 Gut microbiome of native Arab Kuwaitis	16S rRNA gene sequencing	Illumina MiSeq	• Kuwait University, Kuwait	*Bacteroides* was the dominant genus in the gut microbiome of native Arab Kuwaiti adults, with *B. dorei/vulgatus* as the most prevalent phylogenetic group.
**41**	Masoodi et al., 2020 Microbial dysbiosis in irritable bowel syndrome: a single-center metagenomic study in Saudi Arabia	Shotgun metagenomic sequencing	Illumina GAIIx and HiSeq 2000	• King Abdulaziz City for Science and Technology, Saudi Arabia	Significant microbiota differences were observed between irritable bowel syndrome (IBS) patients and controls, with four genera enriched in Saudi IBS patients, findings consistent with studies from other populations.
**42**	Moubareck et al., 2020 Profiles of human milk oligosaccharides and their relations to the milk microbiota of breastfeeding mothers in Dubai	16S rRNA gene sequencing	Illumina MiSeq	• Zayed University, United Arab Emirates • Dutch Province of Limburg with a subsidy grant to HEFI	The study characterized human milk oligosaccharide profiles of breastfeeding mothers in Dubai and identified limited correlations with milk microbiota composition.
**43**	Murugesan et al., 2020 Profiling the salivary microbiome of the Qatari population	16S rRNA gene sequencing	Illumina MiSeq	• Sidra medicine internal research fund, Qatar	The Qatari population exhibited distinct microbial profiles compared to other global populations, with salivary microbiome composition influenced by sex, age, oral health, smoking, and coffee consumption.
**44**	Al Bataineh et al., 2020 Revealing links between gut microbiome and its fungal community in type 2 diabetes mellitus among Emirati subjects: a pilot study	16S rRNA gene and internal transcribed spacer (ITS2) gene sequencing	Illumina MiSeq	• University of Sharjah, United Arab Emirates • Boehringer Ingelheim grant • EU litmus grant • Leducq Foundation • JPI-HDHL MICRODIET consortium grant	A pilot study in Emirati individuals showed shifts in gut microbiome composition and function in those with type 2 diabetes compared to non-diabetic controls.
**45**	Singh et al., 2020 The potential role of vitamin D supplementation as a gut microbiota modifier in healthy individuals	16S rRNA gene sequencing	Illumina MiSeq	• Qatar University, Qatar	Vitamin D supplementation in deficient or insufficient healthy females led to favorable changes in gut microbiota composition and diversity, along with improvements in clinical markers of kidney and liver function.
**46**	Khan et al., 2019 Evaluation of gut bacterial community composition and antimicrobial resistome in pregnant and non-pregnant women from Saudi population	16S rRNA gene sequencing	Illumina MiSeq	• King Abdulaziz City for Science and Technology, Saudi Arabia	Pregnancy-related changes in gut microbiota may support maternal health, but increased antibiotic resistance poses potential risks to both mother and fetus.
**47**	Sohail et al., 2019 Profiling the oral microbiome and plasma biochemistry of obese hyperglycemic subjects in Qatar	16S rRNA gene sequencing	Illumina MiSeq	• Qatar National Research Fund, Qatar	While overall oral microbiome diversity did not differ between groups, obese individuals showed a higher *Firmicutes/Bacteroidetes* ratio, a trait linked to obesity.
**48**	Shami et al., 2019 The prevalence of the culturable human skin aerobic bacteria in Riyadh, Saudi Arabia	16S rRNA gene sequencing	No mention	• Princess Nourah bint Abdulrahman University, Saudi Arabia	Bacterial diversity varied by age and sex, with higher diversity observed in the elderly. The foot had the highest microbial richness, followed by the hand and scalp.
**49**	Madi et al., 2018 Metagenomic analysis of viral diversity in respiratory samples from patients with respiratory tract infections in Kuwait	Multiplex real-time PCR and whole transcriptome sequencing	Illumina MiSeq	• Kuwait University, Kuwait	Metagenomic sequencing demonstrated greater sensitivity than RT-Real-Time PCR in detecting respiratory viruses, suggesting its potential as a diagnostic tool in clinical virology.
**50**	Alomair et al., 2018 Colonic mucosal microbiota in colorectal cancer: a single-center metagenomic study in Saudi Arabia	16S rRNA gene sequencing	Illumina HiSeq	• King Abdulaziz City for Science and Technology, Saudi Arabia	The study provided insight into the gut microbiota of colorectal cancer patients in Saudi Arabia, identifying several genera significantly enriched compared to controls.
**51**	Vallès et al., 2018 Types of tobacco consumption and the oral microbiome in the United Arab Emirates Healthy Future (UAEHFS) Pilot Study	16S rRNA gene sequencing	Illumina (authors did not mention which sequencing platform)	• New York University Abu Dhabi Research Institute, United Arab Emirates	Cigarettes and local tobacco products were found to alter oral microbiome structure and specific taxa abundance in the Emirati population.
**52**	Al-Rezaki et al., 2017 Assessment of the microbiome collected from the reproductive tracts of women from Saudi Arabia and its potential influence on infertility	tDNA-PCR (transfer DNA PCR) fingerprinting	No mention	• King Abdulaziz City for Science and Technology, Saudi Arabia	Vaginal microbiota composition varied by pH range, with *Lactobacillus* species dominant at pH 3.0–4.5, and a shift toward *Gardnerella, Peptostreptococcus*, and other anaerobes observed at higher pH levels.
**53**	Angelakis et al., 2016 Gut microbiome and dietary patterns in different Saudi populations and monkeys	16S rRNA gene sequencing	Illumina MiSeq	• King Abdulaziz University, Saudi Arabia	Baboons were found to have enterotypes similar to humans, and microbial richness was lower in populations consuming a Western diet compared to those with traditional diets.
**54**	Yasir et al., 2015a Comparison of the gut microbiota of people in France and Saudi Arabia	16S rRNA gene sequencing	Illumina MiSeq	• King Abdulaziz University, Saudi Arabia	Gut microbiota differences associated with obesity varied between French and Saudi populations, highlighting the influence of population-specific factors on microbiota-obesity associations.

**Table 2A:** Data extracted from eligible animal microbiome studies conducted in the Gulf Cooperation Council (GCC) countries and included in the current systematic review (*n* = 12).

Study no.	Citation and study title	Publication year	Country, sample origin	Study design	Sample type	Sample size	Age	Study period
**1**	Rahmeh et al., 2025 The microbiome and antibiotic resistance in pasteurized camel milk	2025	Kuwait	Observation, cross section	Pasteurized camel milk	*N* = 40	No mention	No mention
**2**	Ramadan et al., 2025 Molecular analysis of bacterial communities in marbled spinefoot (*Siganus rivulatus*) and squaretail coral grouper (*Plectropomus areolatus*), in Jeddah, Saudi Arabia	2025	Saudi Arabia	Observation, cross section	Fish: skin, tissues, gills, and intestine	*N* = 20 from 2 fish species	No mention	No mention
**3**	Rahmeh et al., 2024 Camel milk resistome in Kuwait: genotypic and phenotypic characterization	2024	Kuwait	Observation, cross section	Raw dromedary camel milk	*N* = 8 pooled raw dromedary camel milk	No mention	No mention
**4**	Perveen et al., 2023 Virome diversity of *Hyalomma dromedarii* ticks collected from camels in the United Arab Emirates	2023	United Arab Emirates	Observation, cross section	*H. dromedarii* ticks	*N* = 150 male and female ticks were collected from 15 camels	No mention	June 2021
**5**	Elbir and Alhumam, 2022 Sex differences in fecal microbiome composition and function of dromedary camels in Saudi Arabia	2022	Saudi Arabia	Observation, cross section	Stool	*N* = 20 *M, n* = 10 *F, n* = 10	Mean age: 6 years	April 2021
**6**	Perveen et al., 2022 Assessing temporal changes in microbial communities in *Hyalomma dromedarii* collected from camels in the UAE Using high-throughput sequencing	2022	United Arab Emirates	Observation, cross section	Partially engorged females of *H. dromedarii*	Ticks were collected from 25 camels	Adults	March 2019–February 2020
**7**	Baeshen et al., 2022 Metagenomic analysis of the gut bacteriome of Usherhopper, *Poekilocerus bufonius* (Klug) from Hadda, Saudi Arabia	2022	Saudi Arabia	Observation, cross section	Gut parts from Usherhopper, *Poekilocerus bufonius*	No mention	No mention	August 2014
**8**	Alatawy et al., 2021 Gut microbiome of two different honeybee workers subspecies in Saudi Arabia	2021	Saudi Arabia	Observation, cross section	Honeybee workers of *Apis mellifera jemenitica* and *Apis mellifera carnica*	*N* = 5 samples (each from honeybee workers)	No mention	November 2019
**9**	Alreshidi, 2020 Description of microbial diversity associated with ticks *Hyalomma dromedarii* (Acari: Ixodidae) isolated from camels in Hail region (Saudi Arabia) using massive sequencing of 16S rDNA	2020	Saudi Arabia	Observation, cross section	Ticks samples collected from healthy camels	*N* = 200 ticks	Adults	No mention
**10**	Badger-Emeka et al., 2020 Vitamin D supplementation in laboratory-bred mice: an *in vivo* assay on gut microbiome and body weight	2020	Saudi Arabia	Experimental	Stool	*N* = 20 BALB/C male mice	8–12 weeks	No mention
**11**	Al-Marzooqi et al., 2020 Diversity of intestinal bacterial microbiota of indigenous and commercial strains of chickens using 16s rDNA-based analysis	2020	Oman	Experimental	Luminal content: duodenum, jejunum, ileum, and cecum	*N* = 150 chicks from each strain	0–35 days	No mention
**12**	Al-Asmakh et al., 2020 The effects of gum acacia on the composition of the gut microbiome and plasma levels of short-chain fatty acids in a rat model of chronic kidney disease	2020	Qatar	Experimental	Stool and blood	*N* = 24 male rats	9–10 weeks	No mention

M, Male; F, Female.

**Table 2B:** Data extracted from eligible animal microbiome studies conducted in the Gulf Cooperation Council (GCC) countries and included in the current systematic review (*n* = 12).

Study no.	Citation and study title	Method	Platform	Sponsor	Summary of findings
**1**	Rahmeh et al., 2025 The microbiome and antibiotic resistance in pasteurized camel milk	Shotgun metagenomic sequencing	MGI DNBSEQ-G400 i	• ICGEB and COMSTECH (Committee on Scientific and Technological Cooperation of the Organization of Islamic Cooperation) • The Kuwait Institute for Scientific Research, Kuwait	Antibiotic resistance genes in pasteurized camel milk may be transferable due to proximity to mobile genetic elements, raising food safety concerns and emphasizing the need for improved milk processing strategies.
**2**	Ramadan et al., 2025 Molecular analysis of bacterial communities in marbled spinefoot (*Siganus rivulatus*) and squaretail coral grouper (*Plectropomus areolatus*), in Jeddah, Saudi Arabia	16S rRNA gene sequencing	No mention	• King Abdulaziz University, Saudi Arabia	Distinct microbial profiles were observed between two marine species and across anatomical sites, highlighting variability in richness and composition and the importance of monitoring bacterial contamination for seafood safety.
**3**	Rahmeh et al., 2024 Camel milk resistome in Kuwait: genotypic and phenotypic characterization	Shotgun metagenomic sequencing	MGI DNBSEQ-G400 i	• ICGEB and COMSTECH (Committee on Scientific and Technological Cooperation of the Organization of Islamic Cooperation) • The Kuwait Institute for Scientific Research, Kuwait	The study highlights human health risks associated with raw camel milk and underscores the need for improved hygiene in farms and retail settings to reduce the spread of antibiotic resistance genes.
**4**	Perveen et al., 2023 Virome diversity of *Hyalomma dromedarii* ticks collected from camels in the United Arab Emirates	RNA sequencing	Illumina, NovaSeq 6000 and Oxford Nanopore Technology, MinION	• United Arab Emirates University, United Arab Emirates	The study characterized the RNA virome of *H. dromedarii* ticks associated with camels in the UAE, enhancing understanding of viral diversity in this tick species.
**5**	Elbir and Alhumam, 2022 Sex differences in fecal microbiome composition and function of dromedary camels in Saudi Arabia	16S rRNA gene sequencing	Illumina MiSeq	• King Faisal University, Saudi Arabia	Sex-based differences were observed in camel fecal microbiomes, with females showing enrichment in energy production and cell structure functions, while males had higher levels of amino acid and lipid metabolism pathways.
**6**	Perveen et al., 2022 Assessing temporal changes in microbial communities in *Hyalomma dromedarii* collected from camels in the UAE Using high-throughput sequencing	16S rRNA gene sequencing	Illumina MiSeq	• The United Arab Emirates University, United Arab Emirates	This study reported, for the first time in the UAE, temporal shifts in microbial communities of *H. dromedarii* ticks, offering insights into microbial interactions and potential roles in disease development.
**7**	Baeshen et al., 2022 Metagenomic analysis of the gut bacteriome of Usherhopper, *Poekilocerus bufonius* (Klug) from Hadda, Saudi Arabia	16S rRNA gene sequencing	Illumina HiSeq/MiSeq 2000	• None	*Proteobacteria* was the dominant phylum in *P. bufonius*, with bacterial community composition likely shaped by dietary preferences, including consumption of *R. stricta*.
**8**	Alatawy et al., 2021 Gut microbiome of two different honeybee workers subspecies in Saudi Arabia	16S rRNA gene sequencing	Illumina MiSeq	• Beekeeper Cooperative Association-Al Baha, Saudi Arabia	This first of its kind study in Saudi Arabia showed that the gut microbiota composition of honeybee workers varied by their monophyletic origin, as revealed by high-throughput 16S rRNA sequencing.
**9**	Alreshidi, 2020 Description of microbial diversity associated with ticks *Hyalomma dromedarii* (Acari: Ixodidae) isolated from camels in Hail region (Saudi Arabia) using massive sequencing of 16S rDNA	16S rRNA gene sequencing	Illumina MiSeq	• University of Hail, Saudi Arabia	Ticks from the studied areas harbored diverse microbial communities, with potential future relevance to veterinary and medical fields.
**10**	Badger-Emeka et al., 2020 Vitamin D supplementation in laboratory-bred mice: an *in vivo* assay on gut microbiome and body weight	Bacteria colony count (microbial colonies were identified phenotypically)	Vitek 2 Compact automated system (BioMerieux)	• None	Different doses of vitamin D supplementation in mice affected body weight and gut microbiota composition, suggesting a potential link between vitamin D levels, microbial balance, and weight regulation.
**11**	Al-Marzooqi et al., 2020 Diversity of intestinal bacterial microbiota of indigenous and commercial strains of chickens using 16s rDNA-based analysis	16S rRNA gene sequencing	Illumina MiSeq	• Sultan Qaboos University, Oman	Significant differences in bacterial relative abundance were observed across intestinal segments in two strains, indicating region-specific microbial communities.
**12**	Al-Asmakh et al., 2020 The effects of gum acacia on the composition of the gut microbiome and plasma levels of short-chain fatty acids in a rat model of chronic kidney disease	16S rRNA gene sequencing	Illumina MiSeq	• Qatar University, Qatar	Gum acacia supplementation mitigated gut microbiome alterations, reduced short-chain fatty acids disruptions, and improved renal biomarkers in chronic kidney disease-induced rats, suggesting a microbiome-mediated link between uremia and renal function.

**Table 3A:** Data extracted from eligible environmental microbiome studies conducted in the Gulf Cooperation Council (GCC) countries and included in the current systematic review (*n* = 44).

Study no.	Citation and study title	Publication year	Country	Study design	Sample type	Sample size	Study period
**1**	Fakhraldeen et al., 2024 Shotgun metagenomics reveals the interplay between microbiome diversity and environmental gradients in the first marine protected area in the Northern Arabian Gulf	2025	Kuwait	Observational, cross section	Surface seawater	Multiple samples across four sites	November 2019–February 2020
**2**	Chibwe et al., 2024 Intra-hospital microbiome variability is driven by accessibility and clinical activities	2024	Qatar	Observational, cross section	Surface swab	*N* = 197 NICU, *n* = 37 Additional hospital areas, *n* = 160	Samples were collected prior to full operation in December 2017 and during full operation in March 2018
**3**	Maurice et al., 2024 Networking the desert plant microbiome, bacterial and fungal symbionts structure and assortativity in co-occurrence networks	2024	Saudi Arabia	Observational, cross section	Rhizosphere and soil	Roots and rhizospheres of 25 individuals per plant species were sampled (six plant species were tested)	In August 2021 and in March 2022
**4**	Bamigbade et al., 2024 Gut microbiota modulation, prebiotic and bioactive characteristics of date pomace polysaccharides extracted by microwave-assisted deep eutectic solvent	2024	United Arab Emirates	Experimental	Freshly prepared wet date pomace was obtained from the Al-Foah Date Processing Industry	20 kg (freshly prepared wet date)	No mention
**5**	Al-Tarshi et al., 2024 Bacterial communities across multiple ecological niches (water, sediment, plastic, and snail gut) in mangrove habitats	2024	Oman	Observational, cross section	Sediment, water, snails, and plastics were collected from each Sawadi and Qurum lagoon transects	Sediment, *n* = 3 Water, *n* = 3 Snail gut, *n* = 10 Plastic: unclear	July 2023
**6**	Habibi et al., 2024 Diversity analysis of fungi distributed in inhalable and respirable size fractions of aerosols: a report from Kuwait	2024	Kuwait	Observational, cross section	Aerosol (air samples from different particle size fractions)	*N* = 30 (23 successfully sequenced)	No mention
**7**	Jayasree Subhash et al., 2024 Characterizing date seed polysaccharides: a comprehensive study on extraction, biological activities, prebiotic potential, gut microbiota modulation, and rheology using microwave-assisted deep eutectic solvent	2024	United Arab Emirates	Experimental	Date seeds	50 kg (whole date seeds)	No mention
**8**	Al-Hadidi et al., 2024 The effect of type and combination of fertilizers on eukaryotic microbiome of date palm rhizosphere	2024	Qatar	Experimental	Rhizosphere soil from date palms	*N* = 103	March 2021
**9**	Yaish et al., 2024 The effects of salinity and genotype on the rhizosphere microbiomes in date palm seedlings	2024	Oman	Experimental	Rhizosphere soil from date palm seedlings	Unclear	No mention
**10**	Mousa et al., 2024a High-throughput sequencing reveals the structure and metabolic resilience of desert microbiome confronting climate change	2024	United Arab Emirates	Observational, cross section	Four desert plants native to the UAE desert: *Halocnemum strobilaceum, Panicum turgidum, Haloxylon persicum*, and *Arnebia hispidissima*	*N* = 40 Rhizosphere, *n* = 20 From inside the root tissues of the studied plants, *n* = 20	No mention
**11**	Mousa et al., 2024b The design and development of EcoBiomes: multi-species synthetic microbial consortia inspired by natural desert microbiome to enhance the resilience of climate-sensitive ecosystems	2024	United Arab Emirates	Experimental	Isolate individual microbes from four desert plants native to the UAE desert: *Halocnemum strobilaceum, Panicum turgidum, Haloxylon persicum*, and *Arnebia hispidissima*	Bacterial isolates, *n* = 75	October 2022
**12**	Maurice et al., 2023 Fertility islands, keys to the establishment of plant and microbial diversity in a highly alkaline hot desert	2023	Saudi Arabia	Observational, cross section	Soil	*N* = 32	No mention
**13**	Johar et al., 2023 Wastewater-based epidemiology for tracking bacterial diversity and antibiotic resistance in COVID-19 isolation hospitals in Qatar	2023	Qatar	Observational, cross section	Wastewater	Samples from four hospitals	May–November 2021
**14**	Maloukh et al., 2023 Metagenomic analysis of the outdoor dust microbiomes: a case study from Abu Dhabi, UAE	2023	United Arab Emirates	Observational, cross section	Outdoor dust	*N* = 18	No mention
**15**	Baeshen et al., 2023 Composition, abundance, and diversity of the soil microbiome associated with the halophytic plants tamarix aphylla and halopeplis perfoliata on Jeddah seacoast, Saudi Arabia	2023	Saudi Arabia	Observational, cross section	Rhizosphere and crust soil samples were collected from two plant species *Tamarix aphylla* and *Halopeplis perfoliata*, and one control (from no plant growth)	*N* = 5	July 2020
**16**	Abumaali et al., 2023a Bacterial community structure and predicted function in the rhizosphere of wild and cultivated date palms: effects of fertilizers on composition and functionality	2023	Qatar	Observational, cross section	Rhizosphere soil of wild and cultivated date palms	*N* = 103	No mention
**17**	El-Malah et al., 2023 Marine bacterial community structures of selected coastal seawater and sediment sites in Qatar	2023	Qatar	Observational, cross section	Coastal seawater and sediment	*N* = 20 Seawater, *n* = 10 Sediment, *n* = 10 were taken from five locations	In July and in September 2019
**18**	Habibi et al., 2023 Metagenomes from coastal sediments of Kuwait: insights into the microbiome, metabolic functions and resistome	2023	Kuwait	Observational, cross section	Coastal sediment samples	*N* = 12	September–October 2021
**19**	Alsaedi et al., 2023 Comparison of endophytic microbiome community and rhizosphere in the desert plant *Senna italica*	2023	Saudi Arabia	Observational, cross section	Soil, leaf, and root samples	*N* = 10 *n* = 3 root *n* = 3 leaf *n* = 3 rhizosphere *n* = 1 control soil	April 2021
**20**	Fakhraldeen et al., 2023 Diversity and spatiotemporal variations in bacterial and archaeal communities within Kuwaiti territorial waters of the Northwest Arabian Gulf	2023	Kuwait	Observational, cross section	Seawater samples were collected from three different locations	*N* = 36	September 2019–February 2020
**21**	Yasir et al., 2023a Metagenomic insights into the microbiome and resistance genes of traditional fermented foods in Arabia	2023	Saudi Arabia	Observational, cross section	Dairy fermented foods and non-dairy mixed vegetable pickles	*N* = 11 Dairy, *n* = 9 Pickles, *n* = 2	No mention
**22**	Skariah et al., 2023 Soil properties correlate with microbial community structure in Qatari arid soils	2023	Qatar	Observational, cross section	Soil samples from diverse arid habitats	*N* = 26 from 13 habitats	May 2017
**23**	Baz et al., 2022 Predicted functional shifts due to type of soil microbiome and watering of two wild plants in Western region of Saudi Arabia	2022	Saudi Arabia	Experimental	Rhizosphere and soil	No mention	No mention
**24**	Alotaibi et al., 2022 Metagenomic analysis of bacterial communities of Wadi Namar Lake, Riyadh, Saudi Arabia	2022	Saudi Arabia	Observational, cross section	Water from Wadi Namar Lake	5 Liters	May 2020
**25**	Sulieman et al., 2022 Different ecological, medical, and industrial important bacteria harboring the soil of Hail, Kingdom of Saudi Arabia	2022	Saudi Arabia	Observational, cross section	Soil	*N* =7	No mention
**26**	Ismail and Almutairi, 2022 Bacterioplankton community profiling of the surface waters of Kuwait	2022	Kuwait	Observational, cross section	Surface water samples at 1 m depth were collected from five sites twice	*N* = 10 (5 sites × 2 time points)	In October 2018 and in April 2019
**27**	Jalal et al., 2022 The microbiome of *Suaeda monoica* and *Dipterygium glaucum* from Southern corniche (Saudi Arabia) reveals different recruitment patterns of bacteria and archaea	2022	Saudi Arabia	Observational, cross section	Soil samples were collected from Southern corniche in Jeddah, Saudi Arabia	*N* = 5	No mention
**28**	Alown et al., 2021 Genotypic characterization of soil bacteria in the umm Al-Namil island, Kuwait	2021	Kuwait	Observational, cross section	Soil samples from five regions of Umm Al-Namil Island	*N* = 25	March 2019
**29**	Khan and Khan, 2020 Microbial communities and their predictive functional profiles in the arid soil of Saudi Arabia	2020	Saudi Arabia	Observational, cross section	Soil	*N* = 7	January–February 2019
**30**	Baeshen et al., 2020 Diversity profiling of associated bacteria from the soils of stress tolerant plants from seacoast of Jeddah, Saudi Arabia	2020	Saudi Arabia	Observational, cross section	Soil	*N* = 4	January 2019
**31**	Khan et al., 2020 Rhizosphere microbiome of arid land medicinal plants and extra cellular enzymes contribute to their abundance	2020	Oman	Observational, cross section	Rhizosphere of *Adenium obesum, Aloe dhufarensi*, and *Cleome austroarabica*	For each plant species, 30 soil samples of root rhizosphere regions	June 2016
**32**	Alotaibi et al., 2020 Microbial diversity of some sabkha and desert sites in Saudi Arabia	2020	Saudi Arabia	Observational, cross section	Soil samples from nine geographical locations (sabkha and desert soils)	*N* = 45 (9 sites × 5 subsamples each)	No mention
**33**	Al-Quwaie, 2020 Bacterial community dynamics with rhizosphere of *Calotropis procera* and *Senna alexandrina* desert plants in Saudi Arabia	2020	Saudi Arabia	Observational, cross section	Rhizosphere soil	*N* = 5 *Calotropis procera, n* = 2 *Senna. alexandrina, n* = 2 Bulk soil, *n* = 1	January 2019
**34**	Abed et al., 2019 Habitat-dependent composition of bacterial and fungal communities in biological soil crusts from Oman	2020	Oman	Observational, cross section	Soil crusts	*N* = 45 (triplicates from multiple habitats at six sites)	January 2016
**35**	Al Salameen et al., 2020 Spatiotemporal variations in bacterial and fungal community associated with dust aerosol in Kuwait	2020	Kuwait	Observational, cross section	Airborne dust aerosol particles from remote and urban sites	*N* = 32 Bacteria, *n* = 16 and fungi, *n* = 16 collected over five time points across three seasons (fall, spring, summer) at two sites	October 2017 April 2018 June 2018 July 2018 August 2018
**36**	Aalismail et al., 2019 Functional metagenomic analysis of dust-associated microbiomes above the Red Sea	2019	Saudi Arabia	Observational, cross section	Dust filters samples	Unclear	December 2015–November 2016
**37**	Yasir et al., 2019 Culturomics-based taxonomic diversity of bacterial communities in the hot springs of Saudi Arabia	2019	Saudi Arabia	Observational, cross section	Sediment samples from hot springs (Al-Lith and Jazan)	*N* = 536 purified isolates (six sites)	September 2014–May 2015
**38**	Quoreshi et al., 2019 Untangling the bacterial community composition and structure in selected Kuwait desert soils	2019	Kuwait	Observational, cross section	Rhizosphere and soil	*N* = 8	No mention
**39**	Osman et al., 2016 Bacterial rhizosphere biodiversity from several pioneer desert sand plants near Jizan, Saudi Arabia	2016	Saudi Arabia	Observational, cross section	Rhizosphere and soil	*N* = 5 Rhizosphere, *n* = 4 Surface sand, *n* = 1	March 2012
**40**	Yaish et al., 2016 The use of high throughput DNA sequence analysis to assess the endophytic microbiome of date palm roots grown under different levels of salt stress	2016	Oman	Experimental	Root endophytic microorganisms from *Phoenix dactylifera*	*N* = 24 Control, *n* = 12 Treated, *n* = 12	No mention
**41**	Alzubaidy et al., 2016 Rhizosphere microbiome metagenomics of gray mangroves (*Avicennia marina*) in the Red Sea	2015	Saudi Arabia	Observational, cross section	Rhizosphere sediment and 13 bulk soil sediment	*N* = 6 Rhizosphere, *n* = 4 Bulk soil, *n* = 2	December 2011
**42**	Yasir et al., 2015b Composition of soil microbiome along elevation gradients in Southwestern highlands of Saudi Arabia	2015	Saudi Arabia	Observational, cross section	Soil samples along elevation gradients	*N* = 10 (at different altitudes)	May 2012
**43**	Al-Sarawi et al., 2008 Pyruvate-utilizing bacteria as potential contributors to the food web in the Arabian Gulf	2008	Kuwait	Experimental	Coastal sea water samples (10 replicates) were collected from four different sites	Unclear	In July and October 2004 and in April 2005
**44**	Al-Sayed et al., 2005 Bacterial community and some physico-chemical characteristics in a subtropical mangrove environment in Bahrain	2005	Bahrain	Observational, cross section	Water samples from six stations along a tidal mangrove channel	Unclear	November 1993–March 1994

NICU, Neonatal Intensive Care Unit.

**Table 3B:** Data extracted from eligible environmental microbiome studies conducted in the Gulf Cooperation Council (GCC) countries and included in the current systematic review (*n* = 44).

Study no.	Citation and study title	Method	Platform	Sponsor	Summary of findings
**1**	Fakhraldeen et al., 2024 Shotgun metagenomics reveals the interplay between microbiome diversity and environmental gradients in the first marine protected area in the Northern Arabian Gulf	Shotgun metagenomic sequencing	Illumina HiSeq	• Kuwait Institute for Scientific Research, Kuwait • Kuwait National Focal Point, Kuwait	Microbial diversity was greater within the marine protected area than in surrounding waters and was dominated by *Rhodobacteraceae* and *Flavobacteriaceae*. Environmental factors, particularly temperature, salinity, and nutrient levels such as phosphate, played key roles in shaping microbial community structure.
**2**	Chibwe et al., 2024 Intra-hospital microbiome variability is driven by accessibility and clinical activities	16S rRNA gene sequencing	Illumina MiSeq	• Sidra Medicine, Qatar • WashU | McKelvey School of Engineering (McKelvey Engineering) • National Science Foundation Graduate Research Fellowship Program (GRFP)	Hospital environments contained viable and potentially antibiotic-resistant bacteria that varied across time and location. Correlation with 16S sequencing data indicated the importance of monitoring both pathogenic and commensal microbial communities to strengthen infection control and surveillance efforts.
**3**	Maurice et al., 2024 Networking the desert plant microbiome, bacterial and fungal symbionts structure and assortativity in co-occurrence networks	16S rRNA gene sequencing, internal transcribed spacer (ITS2) and 18S rRNA gene sequencing	Illumina NovaSeq 6000	• SoFunLand project, part of the Oasis program funded under the partnership between the RCU (Royal Commission for AlUla) and AFALULA (Agence Française pour le development d'AlUla)	Network analysis demonstrated that bacterial and fungal symbionts play central roles in shaping the structure and connectivity of the global plant microbiome.
**4**	Bamigbade et al., 2024 Gut microbiota modulation, prebiotic and bioactive characteristics of date pomace polysaccharides extracted by microwave-assisted deep eutectic solvent	16S rRNA gene sequencing	No mention (sequencing was performed at BGI, Hong Kong)	• United Arab Emirates University, UAE • Zayed Center for Health Sciences, UAE	MPS demonstrated distinct physicochemical characteristics and showed broad bioactive potential, including antioxidant, antidiabetic, antihypertensive, anticancer, and antimicrobial activities against both Gram-positive and Gram-negative foodborne pathogens.
**5**	Al-Tarshi et al., 2024 Bacterial communities across multiple ecological niches (water, sediment, plastic, and snail gut) in mangrove habitats	16S rRNA gene sequencing	Illumina MiSeq	• Ministry of Higher Education, Research and Innovation, Oman	Microbial communities were similar across sediments, surface water, plastic debris, and snail guts in both lagoons, likely due to comparable environmental conditions including temperature, salinity, total dissolved solids, and electrical conductivity.
**6**	Habibi et al., 2024 Diversity analysis of fungi distributed in inhalable and respirable size fractions of aerosols: a report from Kuwait	Internal transcribed spacer (ITS3 and ITS4) gene sequencing	Illumina HiSeq 2500	• None	Atmospheric fungal communities, including opportunistic pathogens, were detected across inhalable and respirable aerosol fractions with variable abundance. Continuous exposure of humans and other organisms highlights the need to evaluate potential occupational and environmental health risks.
**7**	Jayasree Subhash et al., 2024 Characterizing date seed polysaccharides: a comprehensive study on extraction, biological activities, prebiotic potential, gut microbiota modulation, and rheology using microwave-assisted deep eutectic solvent	16S rRNA gene sequencing	No mention (sequencing was performed at BGI, Hong Kong)	• The United Arab Emirates University, UAE • Zayed Center for Health Sciences, UAE	Microwave-assisted deep eutectic solvent extraction of date seed polysaccharides demonstrated high efficiency and revealed structurally diverse heteropolysaccharide-like compounds with variable molecular weights and monosaccharide compositions.
**8**	Al-Hadidi et al., 2024 The effect of type and combination of fertilizers on eukaryotic microbiome of date palm rhizosphere	18S SSU rRNA gene sequencing	Illumina HiSeq 2500	• Qatar University, Qatar	Rhizosphere microbiome diversity varied according to cultivar, fertilizer type, and dosage. Wild date palms hosted salt- and drought-tolerant eukaryotes with potential for developing biofertilizers suited to arid environments.
**9**	Yaish et al., 2024 The effects of salinity and genotype on the rhizosphere microbiomes in date palm seedlings	Internal transcribed spacer (ITS2) gene sequencing	Illumina MiSeq	• Sultan Qaboos University, Oman	Rhizosphere microbial communities in date palms showed complex responses to salinity stress, with no clear evidence that native epiphytic fungi significantly enhance salt tolerance.
**10**	Mousa et al., 2024a High-throughput sequencing reveals the structure and metabolic resilience of desert microbiome confronting climate change	16S rRNA gene sequencing	Illumina MiSeq	• Al Ain University, UAE	The study demonstrated high microbial diversity and functional potential in Arabian Peninsula desert ecosystems, identifying metabolically active communities adapted to extreme heat, radiation, drought, and salinity, with important ecological roles.
**11**	Mousa et al., 2024b The design and development of EcoBiomes: multi-species synthetic microbial consortia inspired by natural desert microbiome to enhance the resilience of climate-sensitive ecosystems	16S rRNA gene sequencing	No mention	• Sandooq Al Watan organization, UAE	Preliminary findings suggest that EcoBiome-associated microbial communities enhance plant resilience to drought, heat, and salinity, indicating potential applications for climate adaptation and sustainable food production.
**12**	Maurice et al., 2023 Fertility islands, keys to the establishment of plant and microbial diversity in a highly alkaline hot desert	16S rRNA gene sequencing and internal transcribed spacer (ITS4) gene sequencing	Illumina MiSeq	• SoFunLand project, part of the Oasis program funded under the partnership between the RCU (Royal Commission for AlUla) and AFALULA (Agence Française pour le development d'AlUla)	Findings support the fertility island hypothesis, demonstrating that plant establishment is closely associated with soil conditions and that fertile islands harbor distinct microbial diversity and composition compared with bare soil, suggesting their ecological importance for ecosystem function and conservation.
**13**	Johar et al., 2023 Wastewater-based epidemiology for tracking bacterial diversity and antibiotic resistance in COVID-19 isolation hospitals in Qatar	Quantitative PCR array	7500 Real-Time PCR System (Thermo Fisher Scientific)	• Qatar University, Qatar	Sewage water contained high pathogen loads and antibiotic resistance genes, posing potential risks to public health and aquatic ecosystems.
**14**	Maloukh et al., 2023 Metagenomic analysis of the outdoor dust microbiomes: a case study from Abu Dhabi, UAE	16S rRNA gene sequencing	Illumina HiSeq	• Zayed University, UAE	Outdoor environmental samples from the UAE revealed diverse bacterial communities not associated with infectious agents, suggesting their value for characterizing the human microbial exposome, improving pathogen surveillance, and understanding atmospheric and climate-related microbial processes.
**15**	Baeshen et al., 2023 Composition, abundance, and diversity of the soil microbiome associated with the halophytic plants *Tamarix aphylla* and *Halopeplis perfoliata* on Jeddah seacoast, Saudi Arabia	16S rRNA gene sequencing and internal transcribed spacer (ITS2 and ITS5) gene sequencing	Illumina MiSeq	• University of Jeddah, Saudi Arabia	The study identified diverse microbial communities associated with stress-tolerant halophytic plants in coastal soils of southern Jeddah, suggesting their potential role in plant adaptation to saline and harsh environmental conditions.
**16**	Abumaali et al., 2023a Bacterial community structure and predicted function in the rhizosphere of wild and cultivated date palms: effects of fertilizers on composition and functionality	16S rRNA gene sequencing	Illumina MiSeq	• Qatar University, Qatar	Date palm cultivars influenced rhizosphere bacterial community composition across locations, potentially enhancing plant-associated benefits, and provided initial insights into soil bacterial diversity associated with wild date palms.
**17**	El-Malah et al., 2023 Marine bacterial community structures of selected coastal seawater and sediment sites in Qatar	16S rRNA gene sequencing	Illumina MiSeq	• Qatar Environment and Energy Research Institute, Qatar	Microbial composition and density varied by sampling location and sample type, suggesting the importance of spatial and temporal factors in shaping bacterial biodiversity patterns.
**18**	Habibi et al., 2023 Metagenomes from coastal sediments of Kuwait: insights into the microbiome, metabolic functions and resistome	Shotgun metagenomic sequencing	Illumina NovaSeq 6000	• The Kuwait Institute for Scientific Research, Kuwait	Microbial community composition was shaped by environmental conditions and effluent discharge, with spatial variation associated with site-specific contamination levels. The findings suggest the need to incorporate pollution indicators, seasonal variation, and contextual factors when assessing environmental microbial diversity and function.
**19**	Alsaedi et al., 2023 Comparison of endophytic microbiome community and rhizosphere in the desert plant *Senna italica*	16S rRNA gene sequencing	Illumina MiSeq	• University of Jeddah, Saudi Arabia	Endophytic and rhizospheric microbial communities were associated with plant growth and survival under harsh conditions, with distinct dominant taxa identified. The findings suggest potential roles for these microbes as indicators of plant resilience and highlight previously unreported taxa that may contribute to abiotic stress tolerance.
**20**	Fakhraldeen et al., 2023 Diversity and spatiotemporal variations in bacterial and archaeal communities within Kuwaiti territorial waters of the Northwest Arabian Gulf	Shotgun metagenomic sequencing	Illumina HiSeq	• The Kuwait Institute for Scientific Research, Kuwait	This first shotgun metagenomic analysis of bacterial and archaeal communities in the northwest Arabian Gulf established baseline data and highlighted functional genetic adaptations of resident microbes to extreme environmental conditions.
**21**	Yasir et al., 2023a Metagenomic insights into the microbiome and resistance genes of traditional fermented foods in Arabia	16S rRNA gene sequencing and shotgun metagenomic sequencing	Illumina MiSeq	• Ministry of Education, Saudi Arabia	Traditional fermented milk showed distinct bacterial diversity at lower taxonomic levels compared with other fermented dairy and pickle products.
**22**	Skariah et al., 2023 Soil properties correlate with microbial community structure in Qatari arid soils	16S rRNA gene sequencing	Illumina MiSeq	• The Qatar National Research Fund, Qatar	Qatari soils contained diverse habitat-specific microbial communities dominated by *Actinobacteria, Firmicutes*, and *Proteobacteria*. Soil texture influenced microbial diversity, and the abundance of *Actinobacteria* suggests potential for discovering novel bioactive compounds.
**23**	Baz et al., 2022 Predicted functional shifts due to type of soil microbiome and watering of two wild plants in Western region of Saudi Arabia	16S rRNA gene sequencing	No mention	• Princess Nourah bint Abdulrahman University, Saudi Arabia	Aspartyl/glutamyl-tRNA amidotransferase subunit A contributes to plant adaptation to watering conditions by maintaining accurate amino acid–tRNA pairing, which helps reduce oxidative stress and translational errors.
**24**	Alotaibi et al., 2022 Metagenomic analysis of bacterial communities of Wadi Namar Lake, Riyadh, Saudi Arabia	16S rRNA gene sequencing	Illumina MiSeq	• Ministry of Education, Saudi Arabia	The study profiled bacterial community composition in Wadi Namar Lake and generated baseline data to inform environmental protection and ecosystem restoration efforts.
**25**	Sulieman et al., 2022 Different ecological, medical, and industrial important bacteria harboring the soil of Hail, Kingdom of Saudi Arabia	16S rRNA gene sequencing	No mention	• University of Hail, Saudi Arabia	Soil from the Hail region harbored diverse and resilient bacterial communities adapted to harsh environmental conditions, with ecological importance and potential for beneficial biotechnological applications.
**26**	Ismail and Almutairi, 2022 Bacterioplankton community profiling of the surface waters of Kuwait	16S rRNA gene sequencing	Illumina MiSeq	• Kuwait University, Kuwait	Bacterioplankton communities in Kuwait waters showed clear temporal and spatial variation, with strong seasonal influences from natural and anthropogenic stressors shaping microbial composition and predicted functional profiles.
**27**	Jalal et al., 2022 The microbiome of *Suaeda monoica* and *Dipterygium glaucum* from Southern corniche (Saudi Arabia) reveals different recruitment patterns of bacteria and archaea	16S rRNA gene sequencing	Illumina MiSeq	• Princess Nourah bint Abdulrahman University, Saudi Arabia	Soil microbiomes were dominated by major bacterial phyla, with plant-associated soils showing higher abundance of specific bacteria and archaea than non-vegetated soils, indicating plant-driven shifts in microbial composition.
**28**	Alown et al., 2021 Genotypic characterization of soil bacteria in the umm Al-Namil Island, Kuwait	16S rRNA gene sequencing	ABI 3130xl genetic analyzer	• The public authority for applied education and training, Kuwait	*Gammaproteobacteria* and *Bacilli* were the predominant groups, with *Pseudomonas* and *Bacillus* consistently dominant across samples, while potentially pathogenic species were detected at low abundance.
**29**	Khan and Khan, 2020 Microbial communities and their predictive functional profiles in the arid soil of Saudi Arabia	16S rRNA gene sequencing	Illumina HiSeq 2500	• The Zayed University, UAE	Saudi desert microbial communities were dominated by *Actinobacteria, Proteobacteria*, and *Firmicutes*, with key taxa involved in biogeochemical cycling, including *Rhodoplanes, Cyanobacteria*, and nitrogen-fixing *Rhizobium* and *Bradyrhizobium*.
**30**	Baeshen et al., 2020 Diversity profiling of associated bacteria from the soils of stress tolerant plants from seacoast of Jeddah, Saudi Arabia	16S rRNA gene sequencing	Illumina (authors did not mention which sequencing platform)	• University of Jeddah, Saudi Arabia	Rhizospheric microbial communities were identified as potential biomarkers of plant growth and stress tolerance, with applications in developing bio-based strategies to improve agricultural productivity and industrial processes.
**31**	Khan et al., 2020 Rhizosphere microbiome of arid land medicinal plants and extra cellular enzymes contribute to their abundance	16S rRNA gene sequencing and internal transcribed spacer (ITS2 and ITS4) gene sequencing	Illumina MiSeq	• Oman Research Council, Oman	Core rhizosphere microbial communities were identified across three arid plant species, with composition varying by location, host plant, and soil properties. Detected extracellular enzyme activity suggests a role in supporting plant growth and adaptation under harsh environmental conditions.
**32**	Alotaibi et al., 2020 Microbial diversity of some sabkha and desert sites in Saudi Arabia	16S rRNA gene sequencing and 18S rRNA gene sequencing	Automated DNA sequencing system (Applied Biosystems)	• Princess Nourah bint Abdulrahman University, Saudi Arabia	Saudi soil microbial communities exhibited high diversity shaped by physicochemical properties. Fungal diversity exceeded bacterial diversity, indicating greater fungal resilience in extreme environments such as Sabkha and desert ecosystems.
**33**	Al-Quwaie, 2020 Bacterial community dynamics with rhizosphere of *Calotropis procera* and *Senna alexandrina* desert plants in Saudi Arabia	16S rRNA gene sequencing	Illumina MiSeq	• None	Variation in soil microbial abundance was associated with plant resilience to biotic and abiotic stresses, indicating that rhizospheric microbiota may serve as biomarkers of plant growth and survival under harsh environmental conditions.
**34**	Abed et al., 2019 Habitat-dependent composition of bacterial and fungal communities in biological soil crusts from Oman	16S rRNA gene sequencing and internal transcribed spacer (ITS1 and ITS2) gene sequencing	Illumina HiSeq 2500	• Kuwait Foundation for Advancement of Sciences, Kuwait • Kuwait Institute for Scientific Research, Kuwait	Persistent microbial populations were detected in size-fractionated aerosols across Kuwait, with clear spatial and seasonal variation. Bacterial diversity changed by location and season, whereas fungal communities remained relatively stable, and higher richness was observed at remote sites.
**35**	Al Salameen et al., 2020 Spatiotemporal variations in bacterial and fungal community associated with dust aerosol in Kuwait	16S rRNA gene sequencing and internal transcribed spacer (ITS) gene sequencing	Illumina MiSeq	• Sultan Qaboos University, Oman	Bacterial and fungal communities in cyanobacteria- and lichen-dominated desert crusts in Oman were shaped by local soil properties and site-specific environmental conditions.
**36**	Aalismail et al., 2019 Functional metagenomic analysis of dust-associated microbiomes above the Red Sea	Shotgun metagenomic sequencing	Illumina HiSeq 4000	• King Abdullah University of Science and Technology, Saudi Arabia	Sandstorm-associated microbial communities exhibited traits that support long-distance dispersal, indicating their potential to transport diverse microorganisms, including possible pathogens, and to influence interactions among environmental, human, and animal microbiomes.
**37**	Yasir et al., 2019 Culturomics-based taxonomic diversity of bacterial communities in the hot springs of Saudi Arabia	16S rRNA gene sequencing and MALDI-TOF mass spectrometry	ABI Prism Sequencer 3730 (Applied Biosystems, USA)	• King Abdulaziz City for Science and Technology, Saudi Arabia	Saudi Arabian hot springs harbored diverse culturable bacterial communities, including species associated with human activity, suggesting anthropogenic influence. Further characterization is required to better understand the origin and potential implications of these microbial populations.
**38**	Quoreshi et al., 2019 Untangling the bacterial community composition and structure in selected Kuwait desert soils	16S rRNA gene sequencing	Illumina MiSeq	• Kuwait Foundation for the Advancement of Sciences, Kuwait • Kuwait Institute for Scientific Research, Kuwait	This study generated baseline data on bacterial diversity and community structure in Kuwait desert soils, providing a reference for future ecological and environmental investigations.
**39**	Osman et al., 2016 Bacterial rhizosphere biodiversity from several pioneer desert sand plants near Jizan, Saudi Arabia	16S rRNA gene sequencing	Pyrosequencing, Roche/454	• Center National de la Recherche Scientifique (CNRS), France	*Bacteroidetes, Firmicutes*, and *Proteobacteria* dominated bacterial communities in Jizan desert samples, suggesting environmentally influenced microbial diversity relevant to desert and sandstone ecosystems.
**40**	Yaish et al., 2016 The use of high throughput DNA sequence analysis to assess the endophytic microbiome of date palm roots grown under different levels of salt stress	16S rRNA gene sequencing and internal transcribed spacer (ITS3 and ITS4) gene sequencing	GS FLX 454, Roche	• Sultan Qaboos University, Oman	The study provides baseline characterization of endophytic microbial communities in date palm roots and demonstrates their potential role in enhancing salinity tolerance.
**41**	Alzubaidy et al., 2016 Rhizosphere microbiome metagenomics of gray mangroves (*Avicennia marina*) in the Red Sea	Shotgun metagenomic sequencing	Pyrosequencing, 454 GS FLX Titanium technology	• King Abdullah University of Science and Technology, Saudi Arabia • ARAMCO, Saudi Arabia • SABIC, Saudi Arabia	The first metagenomic analysis of Red Sea mangrove ecosystems revealed diverse and functionally rich microbial communities in the rhizosphere and surrounding sediments of *Avicennia marina*, suggesting their ecological importance.
**42**	Yasir et al., 2015b Composition of soil microbiome along elevation gradients in Southwestern highlands of Saudi Arabia	16S rRNA gene sequencing	454 FLX–titanium amplicon pyrosequencing technology (Roche, Basel Switzerland)	• King Abdulaziz University, Saudi Arabia	Soil bacterial diversity and physicochemical properties were not consistently influenced by elevation. A large proportion of shared operational taxonomic units indicated the presence of a core bacterial community across soils in the southwestern highlands of Saudi Arabia.
**43**	Al-Sarawi et al., 2008 Pyruvate-utilizing bacteria as potential contributors to the food web in the Arabian Gulf	16S rRNA gene sequencing	ABI 3130xl genetic analyzer (Applied Biosystems, USA)	• Kuwait University, Kuwait	Surface-water bacteria in the Arabian Gulf predominantly metabolized low-molecular-weight compounds, particularly pyruvate derived from primary producers, indicating its importance in sustaining marine microbial food webs.
**44**	Al-Sayed et al., 2005 Bacterial community and some physico-chemical characteristics in a subtropical mangrove environment in Bahrain	Bacteria colony count (microbial colonies were identified phenotypically)	Total coliforms and fecal coliforms bacteria were counted by standard membrane filter procedures	• None	Mangrove habitats exhibited higher nitrate and phosphate levels than adjacent seawater due to inputs from sewage and agricultural drainage. Although mangroves showed some natural self-purification capacity, anthropogenic pollution was found to adversely affect ecosystem health.

The distribution of microbiome research output across the GCC countries is presented in Figure 2B. Saudi Arabia contributed the largest proportion of studies, accounting for 44% of all included publications. Qatar ranked second, contributing 19%, followed by Kuwait and the United Arab Emirates, which accounted for 16 and 14%, respectively. In contrast, Oman contributed 5% of the studies, while Bahrain had the lowest research output, with only one study (1%). The uneven distribution of research output across the GCC countries likely reflects differences in national research investment, infrastructure capacity, and the presence of established academic and genomic research programs. This disparity may contribute to fragmented regional research development and underscores the need for coordinated strategies to support more balanced scientific capacity across the GCC countries.

Temporal trends in microbiome research across the GCC countries are illustrated in Figure 2C. Human microbiome research was largely absent prior to 2015, with no eligible studies identified between 2005 and 2014. Initial activity emerged gradually, with one human microbiome study published annually between 2015 and 2017. A modest increase was observed in 2018 and 2019, with three studies published each year. Research output increased substantially in 2020, with six human microbiome studies published, reaching a peak in 2021 with 11 publications. This upward trend continued, with 10 studies published in both 2022 and 2023 and seven studies in 2024, reflecting sustained growth and increasing regional interest in human microbiome research.

Animal microbiome research remained limited throughout the study period (Figure 2C). No animal microbiome studies were identified before 2020. The first four studies were published in 2020, followed by one study in 2021. Publication activity increased modestly in 2022 with three studies but declined again in 2023 and 2024, with one study published in each year. Overall, animal microbiome research represented the smallest proportion of microbiome investigations across the GCC countries. Environmental microbiome research emerged earlier than both human and animal studies but remained sporadic during the initial years (Figure 2C). The earliest environmental microbiome studies were published in 2005 and 2008, with one study each. Following a prolonged period of limited activity, research output resumed in 2016 with three publications. A marked increase occurred from 2020 onward, with seven studies published in 2020, five in 2022, and 11 studies in both 2023 and 2024. These temporal trends indicate that microbiome research in the GCC countries is a relatively recent and rapidly expanding field, with accelerated growth observed after 2020. However, the asynchronous development across human, animal, and environmental domains suggests that research efforts have evolved in parallel rather than through integrated One Health approaches. This pattern reinforces the need for coordinated, cross-sectoral research strategies to align emerging efforts and maximize their translational impact.

### 3.4 Distribution of human, animal, and environmental microbiome research across GCC countries

**Figure 3:**
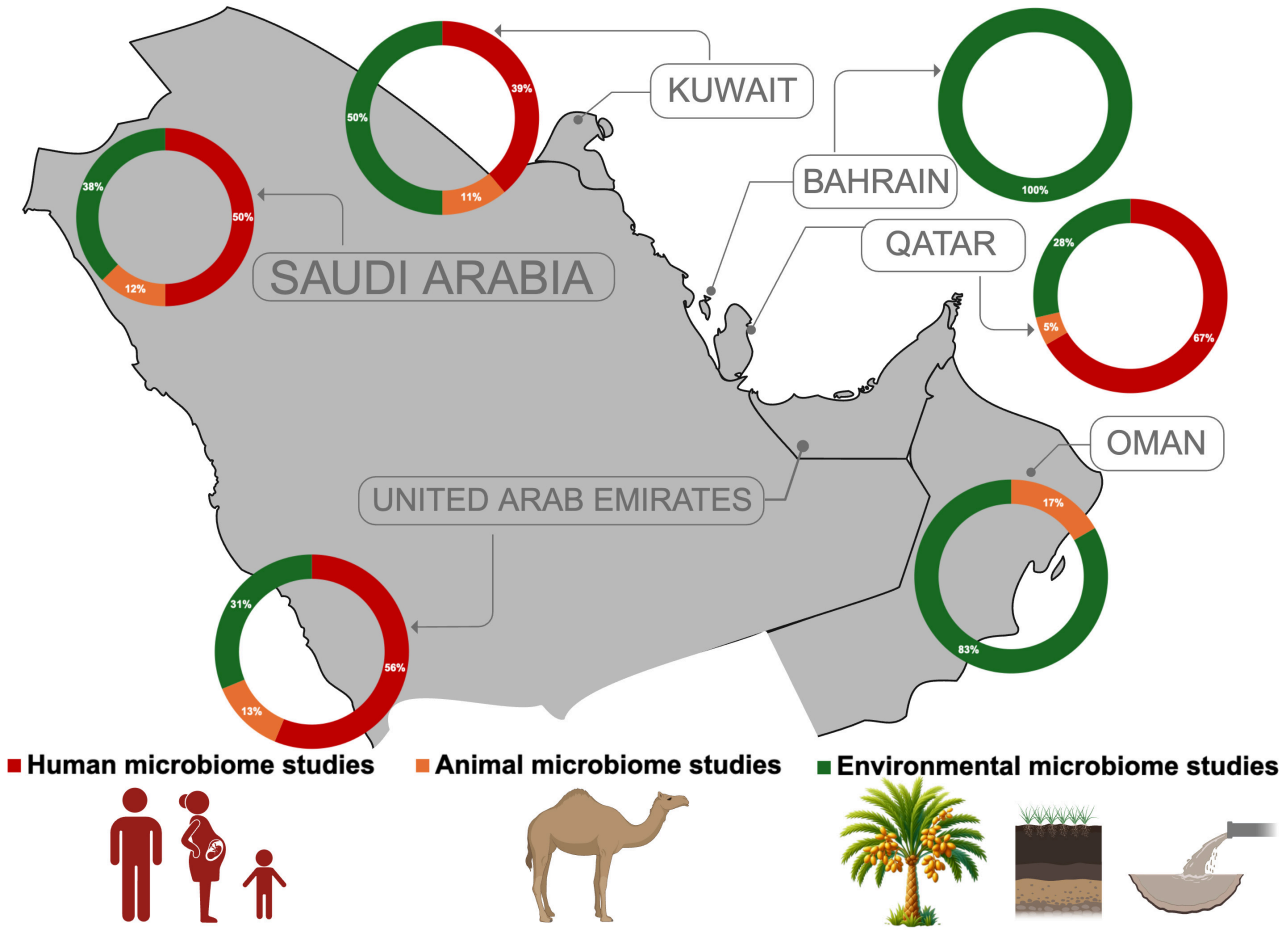
Geographic distribution of microbiome research across Gulf Cooperation Council (GCC) countries by study domain. Each country is annotated with a pie chart showing the proportion of microbiome studies by domain (human, animal, and environmental), illustrating the distribution of microbiome research across the region. The figure was created in BioRender. Aldriwesh, M. (2026) https://BioRender.com/vzpijdv.

We examined the geographical origin of microbiome samples reported in all 110 eligible human, animal, and environmental studies conducted across the GCC countries. The included studies collected samples from Saudi Arabia, Qatar, Kuwait, United Arab Emirates, Oman, and Bahrain (Figure 3). Saudi Arabia contributed the highest number of microbiome studies (*n* = 48). Of these, 50% investigated human microbiomes, 12% focused on animal microbiomes, and 38% examined environmental microbiomes. Qatar ranked second with 21 studies, of which 67% were human microbiome studies, 5% were animal studies, and 28% were environmental microbiome studies. Kuwait contributed 18 microbiome studies, comprising 39% human, 11% animal, and 50% environmental microbiome studies. The United Arab Emirates reported 16 studies, including 56% human, 13% animal, and 31% environmental studies. Oman contributed six microbiome studies, consisting of one animal study (17%) and five environmental studies (83%), with no eligible human microbiome studies identified. Bahrain reported a single microbiome study, which was environmental in origin.

Variation in research focus across countries suggests emerging patterns, with some countries emphasizing human microbiome research (e.g., Saudi Arabia and Qatar), while others show greater engagement in environmental studies (e.g., Oman and Kuwait). This uneven distribution across both geography and research domains further reflects fragmented development of microbiome research in the region and limited cross-sectoral integration. Collectively, these patterns underscore the need for coordinated regional strategies to balance research capacity and promote One Health approaches across the GCC countries.

### 3.5 Study designs, methods, and platforms in human, animal, and environmental microbiome research in GCC countries

**Figure 4:**
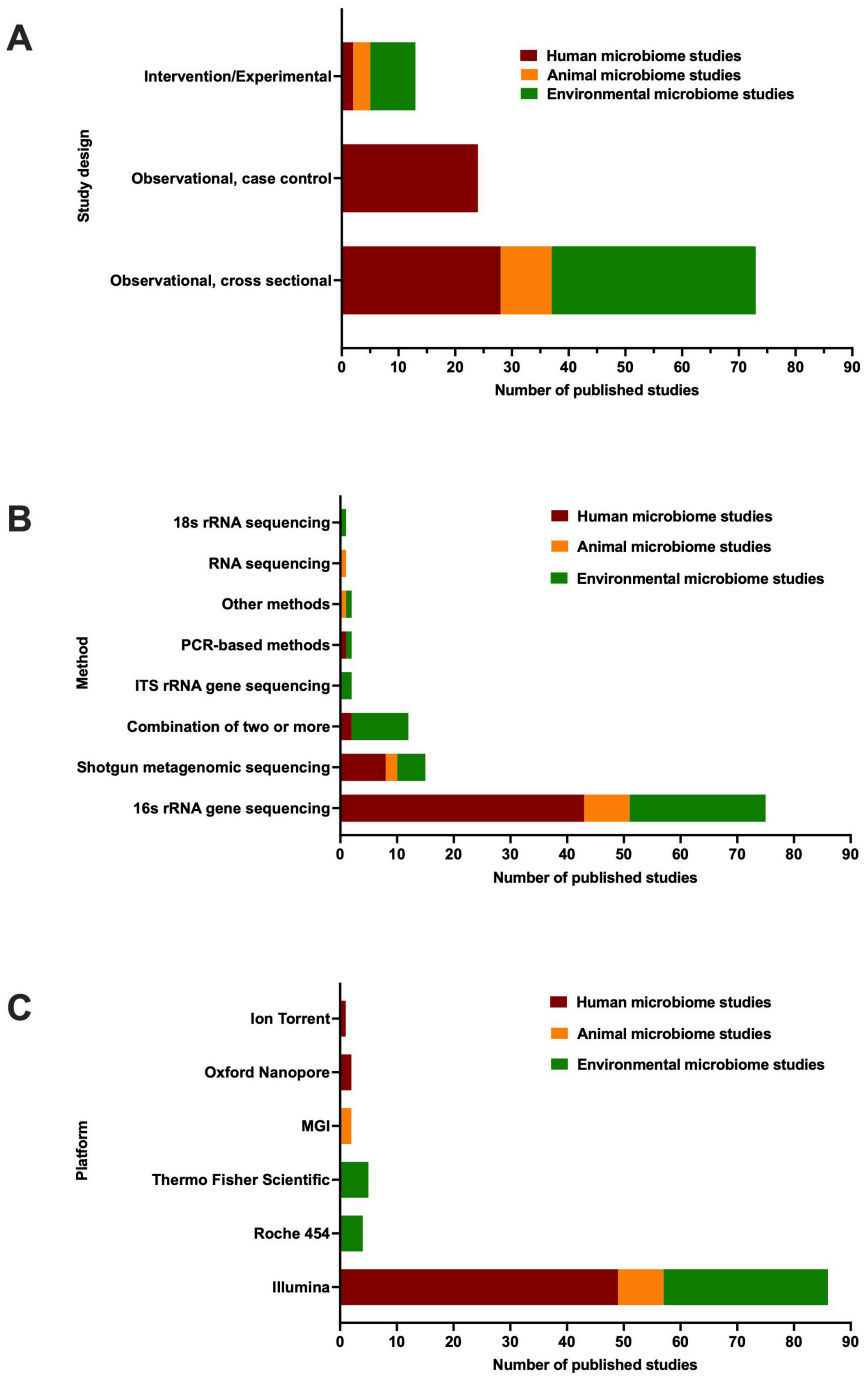
Study designs, analytical methods, and laboratory platforms used in microbiome research across Gulf Cooperation Council (GCC) countries. **(A)** Distribution of study design types by study domain (human, animal, and environmental). **(B)** Laboratory and analytical methods used for microbiome characterization across study domains. Other methods included culture-based approaches: one animal microbiome study (Badger-Emeka et al., 2020) and one environmental microbiome study (Al-Sayed et al., 2005) used bacterial colony counting with phenotypic identification. **(C)** Laboratory platforms and sequencing technologies used for microbiome analysis, grouped by study domain.

Three primary study designs were identified across human, animal, and environmental microbiome research conducted in the GCC countries. Observational cross-sectional studies were the most prevalent design, accounting for 52% of human studies, 75% of animal studies, and 81% of environmental studies. Among human microbiome studies, observational case-control designs were also common, representing 44% of publications. Interventional or experimental designs were less frequently used, comprising 4% of human studies, 25% of animal studies, and 18% of environmental studies (Tables 1A–3A; Figure 4A). These findings indicate that microbiome research in the GCC countries is predominantly based on observational study designs, with limited use of longitudinal or interventional approaches. This reliance may constrain the ability to infer causal relationships and limit the translational potential of microbiome research in clinical, agricultural, and environmental contexts.

With respect to microbiome characterization methods, most studies relied on 16S rRNA gene sequencing. This approach was used in 80% of human studies, 67% of animal studies, and 54% of environmental studies. Shotgun metagenomic sequencing was employed less frequently, reported in 15% of human studies, 17% of animal studies, and 11% of environmental studies. Multiple analytical methods were combined in 4% of human studies and 23% of environmental studies. Other molecular approaches were used less frequently. PCR-based methods were reported in one human study and one environmental study. Among environmental microbiome studies, internal transcribed spacer (ITS) rRNA gene sequencing was used in 4%, and 18S rRNA gene sequencing in 2%. RNA sequencing was reported in one animal study, while traditional methodological approaches were used in one animal study and one environmental study (Tables 1B–3B; Figure 4B).

Microbiome investigation platforms were dominated by Illumina sequencing technologies, which were used in 91% of human studies, 67% of animal studies, and 66% of environmental studies. In human microbiome research, smaller proportions of studies used Oxford Nanopore Technologies (Oxford Nanopore Technologies Ltd., Oxford, United Kingdom) (4%) and Ion Torrent platforms (2%). Among animal microbiome studies, additional platforms included MGI sequencing platforms (17%) and the Vitek 2 Compact automated system (bioMérieux) (8%). Environmental microbiome studies employed a range of analytical platforms, including Roche 454 sequencing (9%), Thermo Fisher Scientific platforms (11%), and traditional culture-based or plate-count methods (2%; Tables 1B–3B; Figure 4C). Information on analytical platforms was not reported in two human studies, one animal study, and five environmental microbiome studies in the original publications.

The widespread use of 16S rRNA gene sequencing further suggests that much of the research remains focused on taxonomic profiling, with relatively limited adoption of more advanced approaches such as shotgun metagenomics, which enable functional and strain-level characterization. While the dominance of Illumina platforms reflects a degree of methodological standardization across the region, the overall methodological landscape indicates an emerging field that has yet to fully adopt advanced multi-omics and integrative analytical approaches. Taken together, these patterns show important methodological gaps that may limit the integration of microbiome research within a One Health framework and underscore the need for investment in longitudinal study designs and multi-domain analytical capacity across the GCC countries.

### 3.6 Body sites, sample types, and diseases studied in human microbiome research in GCC countries

**Figure 5:**
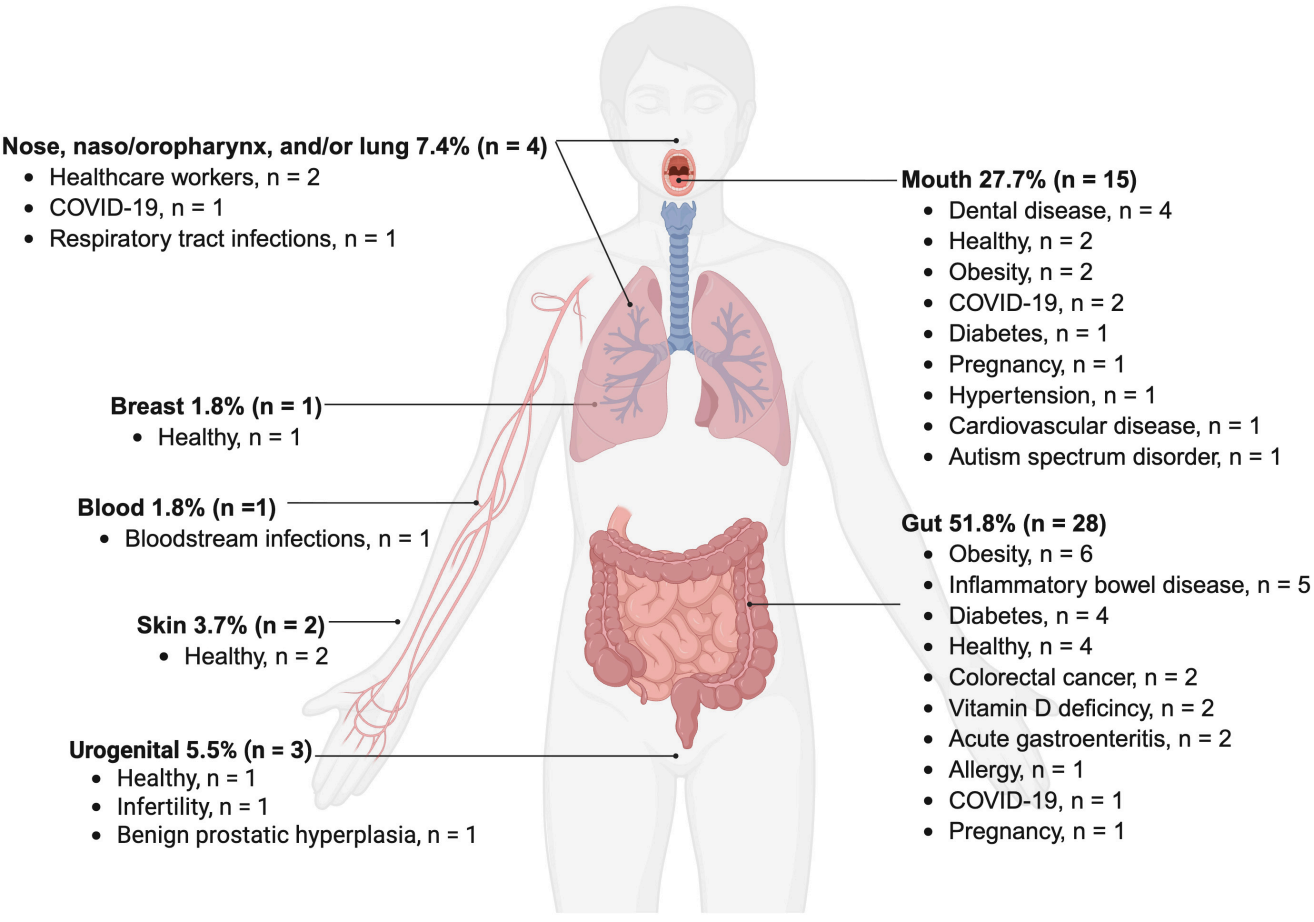
Body sites investigated in human microbiome studies conducted in Gulf Cooperation Council (GCC) countries and associated disease focus. The diseases investigated are presented for each body site, illustrating the distribution of research focus across anatomical sites. The figure was created in BioRender. Aldriwesh, M. (2026) https://BioRender.com/zcqmohp.

Across the 54 eligible human microbiome studies conducted in the GCC countries, the gut was the most frequently investigated body site, accounting for 52% of studies. The oral cavity was the second most studied site, representing 28% of studies, while the breast and blood were the least investigated sites, each represented in 2% of studies. Fecal samples were the most commonly collected sample type, reported in 50% of studies, followed by saliva in 18% and blood in 9%. Skin swabs were used in 8% of studies. All other sample types were reported in a single study each, including respiratory tract samples (e.g., nasopharyngeal swabs, bronchoalveolar lavage, and sputum), anterior nares samples, cervicovaginal fluid, vaginal swabs, urine, colonic mucosa, prostate tissue, gastrointestinal mucosal wash, duodenal mucosal samples, infected intracanal samples, breast milk, subgingival and supragingival plaque, tongue scrapings, and mouthwash samples (Figure 5; Table 1A). These findings indicate that human microbiome research in the GCC countries is primarily focused on a limited number of body sites, particularly the gut and oral microbiomes, with comparatively limited exploration of other clinically relevant niches such as respiratory, urogenital, and tissue-associated microbiomes. This concentration may reflect both global research trends and the relative accessibility of sample types such as fecal and oral samples, but it also suggests potential gaps in understanding microbiome dynamics across diverse body systems.

To assess the relevance of microbiome research to major public health concerns in the GCC countries, the disease focus of included studies was examined. Of the 54 human microbiome studies, 22% did not focus on a specific disease condition (Table 1A). The remaining 78% investigated microbiome associations with defined health conditions. Among these, 15 studies addressed four major diseases of regional public health importance: cardiovascular disease (*n* = 1), diabetes (*n* = 5), COVID-19 (*n* = 4), and inflammatory bowel disease (*n* = 5). Additional disease areas included respiratory tract infections (*n* = 1) and colorectal cancer (*n* = 2). Other health conditions investigated included obesity (*n* = 10), dental diseases (*n* = 4), vitamin D deficiency (*n* = 2), acute gastroenteritis (*n* = 2), pregnancy (*n* = 2), hypertension (*n* = 1), allergy (*n* = 1), bloodstream infection (*n* = 1), infertility (*n* = 1), benign prostatic hyperplasia (*n* = 1), and autism spectrum disorder (*n* = 1; Figure 5). The distribution of disease focus shows an alignment with prevalent regional health challenges, particularly metabolic disorders such as obesity and diabetes, as well as emerging infectious diseases such as COVID-19. However, the overall diversity of disease areas remains limited, and several conditions of regional importance appear underrepresented.

Sample sizes varied substantially across studies. Most studies involved relatively small cohorts, with 76% enrolling fewer than 100 participants, 15% including between 100 and 499 participants, and 9% enrolling larger cohorts ranging from 500 to fewer than 3,000 participants. The majority of studies focused on adult populations (78%), while 20% included pediatric participants. Only one study (2%) included a mixed population of infants, children, and adults. Sex distribution varied across studies. Thirty-four studies reported women participation (mean sample size 164.3 ± 370.3 participants; minimum 2, maximum 1,544), while 30 studies reported men participation (mean sample size 179.8 ± 374.6 participants; minimum 3, maximum 1,432). Some studies were sex-specific, including eight that involved only women participants and four that included only men participants. Notably, 16 studies did not report participant sex (Table 1A). The predominance of small sample sizes, adult-focused cohorts, and incomplete reporting of participant characteristics demonstrate methodological limitations that may affect the generalizability and reproducibility of findings, particularly in the context of translational microbiome research.

### 3.7 Sample types and sources in animal and environmental microbiome studies in GCC countries

**Figure 6:**
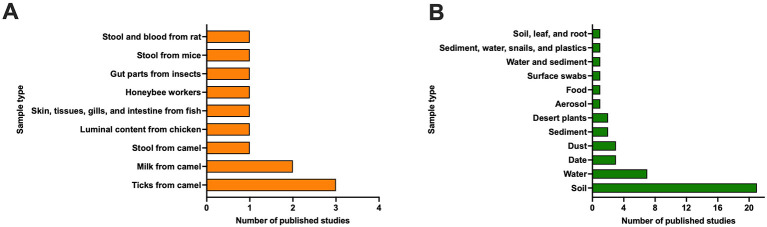
Sample types collected in animal and environmental microbiome studies conducted in Gulf Cooperation Council (GCC) countries. **(A)** Animal microbiome studies categorized by sample type and host species. **(B)** Environmental microbiome studies categorized by sample type.

In animal microbiome studies conducted across the GCC countries, ticks collected from camels were the most frequently investigated sample type, representing 25% of all animal studies. Camel milk was the second most common sample type, accounting for 17% of studies. All other sample types were reported in a single study each (8%), including camel feces, chicken luminal content, and samples from fish (skin, tissues, gills, and intestines). Additional sample sources included honeybee workers, insect gut segments, mouse stool, and rat stool and blood (Figure 6A; Table 3A). These results demonstrate that animal microbiome research in the GCC countries is concentrated on a limited number of species and sample types, particularly camel-associated samples, with comparatively limited investigation of other livestock and wildlife animals relevant to food security and agricultural sustainability.

Among environmental microbiome studies, soil was the predominant sample type, reported in 48% of studies, followed by water samples in 16%. Dates and dust were each analyzed in 7% of studies, while sediments and desert plants were each represented in 4%. Other environmental samples were reported less frequently (2% each), including aerosols, food samples, surface swabs, combined water and sediment samples, snails, plastics, and composite samples consisting of soil, leaves, and roots (Figure 6B; Table 2A). Environmental microbiome research demonstrates broader sample diversity but remains predominantly focused on soil, with comparatively limited attention to other important environmental interfaces such as air, built environments, and aquatic systems.

Together, these patterns suggest that microbiome sampling across animal and environmental domains remains uneven and insufficiently aligned with key One Health interfaces in the region. This emphasizes the need for more comprehensive and integrated sampling strategies that capture microbial interactions across human, animal, and environmental systems in GCC-specific contexts.

### 3.8 Funding sources for microbiome research in GCC countries

**Figure 7:**
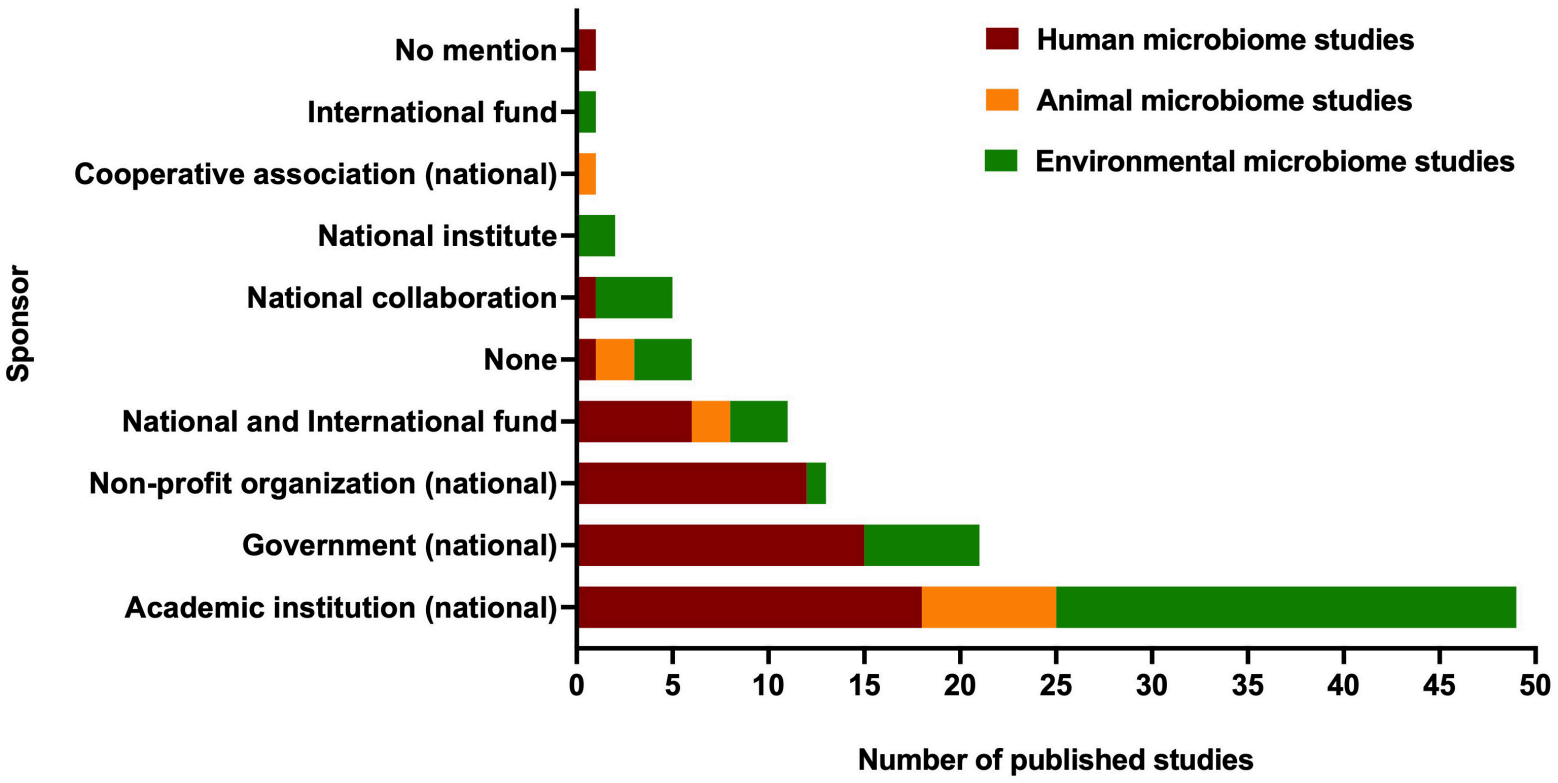
Funding sources supporting microbiome research across Gulf Cooperation Council (GCC) countries. The proportions of human, animal, and environmental microbiome studies are categorized by type of funding source.

Funding sources reported across human, animal, and environmental microbiome studies conducted in the GCC countries were analyzed (Figure 7; Tables 1B–3B). National academic institutions represented the most common source of funding across all study domains, supporting 33% of human studies, 58% of animal studies, and 54% of environmental studies. National government funding was the second most frequently reported source, supporting 28% of human studies and 14% of environmental studies. Nonprofit national organizations provided funding for 22% of human studies and 2% of environmental studies. Combined national and international funding was reported in 11% of human studies, 17% of animal studies, and 7% of environmental studies. A small number of studies reported no funding support, including 2% of human studies, 17% of animal studies, and 7% of environmental studies. National collaborative funding was reported only in human and environmental microbiome research, accounting for 2 and 10% of studies, respectively. National institute funding was reported in environmental microbiome studies (4%), whereas national cooperative association funding was reported only in animal studies (8%). International funding alone was reported in one environmental microbiome study. In addition, one human microbiome study did not specify a funding source.

These findings show that microbiome research in the GCC countries is predominantly supported by national funding sources, with limited engagement in coordinated regional funding mechanisms. While this reflects growing domestic investment, it also suggests a fragmented funding landscape that may constrain cross-country collaboration and large-scale research efforts. These patterns underscore the need for more coordinated and diversified regional funding models, including targeted One Health funding initiatives, to support integrated and translational microbiome research across the GCC countries.

## 4 Discussion

### 4.1 Overview of microbiome research in GCC countries

The microbiome represents one of the most promising frontiers in modern science (Turnbaugh et al., 2007); however, in the GCC countries, its full potential remains underexplored. To our knowledge, this systematic review is the first comprehensive effort to map the microbiome research landscape across all six GCC countries, synthesizing evidence from 110 studies published between 2005 and 2025. Our findings indicate that microbiome research in the region has expanded rapidly in recent years, with a marked increase in publications since 2020, yet research efforts remain largely disconnected, with limited integration across human, animal, and environmental microbiome domains (Figure 2).

Human microbiome studies accounted for nearly half of all publications (49%), followed by environmental microbiome research (40%), whereas animal microbiome studies remained limited (11%). The relatively high proportion of environmental studies likely reflects the region's distinctive ecological characteristics, including extreme desert ecosystems, hypersaline environments, and petroleum-associated habitats that have attracted sustained scientific interest (Alzahrani, 2021). In contrast, the limited representation of animal microbiome research may be associated with underdeveloped veterinary microbiome research capacity and the absence of coordinated livestock surveillance programs integrating microbial profiling. This distribution reflects an imbalance in research priorities across microbiome domains and underscores the need for a more coordinated research agenda.

Geographically, Saudi Arabia contributed the largest share of regional microbiome research output (44%), while Bahrain and Oman together accounted for fewer than 7% of all publications, revealing disparities in research capacity and infrastructure across the GCC countries. Despite growing recognition of the interconnected roles of human, animal, and environmental microbiomes in shaping health and ecosystem resilience, no studies in the region adopted a One Health framework integrating these domains within a single investigation. This absence represents a critical gap in current research efforts and limits the ability to comprehensively understand microbial transmission pathways, antimicrobial resistance dynamics, and cross-species interactions. Addressing this gap will be essential for developing integrated strategies that support disease prevention, environmental monitoring, and sustainable food systems.

The distribution of microbiome research across the GCC countries revealed a landscape characterized by imbalance in both thematic focus and geographic representation. These patterns are not unique to the region. A systematic review of microbiome research conducted in Africa similarly reported fragmented research activity and uneven research capacity across countries (Allali et al., 2021). This observation underscores the global nature of the challenge and points to the need for coordinated strategic investment in microbiome research infrastructure and capacity building. The predominance of human microbiome studies, although understandable given their direct clinical relevance, has occurred alongside comparatively limited attention to animal and environmental microbiomes. Such imbalance may constrain a comprehensive understanding of microbial ecosystems and their interconnected roles across hosts and environments (Trinh et al., 2018). Advancing a more balanced and integrated research portfolio will be critical for enabling One Health approaches that generate actionable insights for public health, agriculture, and environmental management.

Despite significant investments in genomics and laboratory medicine across the GCC countries, a gap remains between microbiome discovery and its clinical or commercial translation. Of the 110 studies identified in this review, most were descriptive, focusing primarily on microbial characterization rather than advancing therapeutic, diagnostic, or applied outcomes (Tables 1–3). This limits the immediate societal impact of microbiome research, as findings are not yet fully translated into interventions that can improve health outcomes or environmental management practices. A key structural barrier is the fragmentation of the regional microbiome research pipeline. Although the GCC countries have invested substantially in state-of-the-art sequencing facilities, persistent operational challenges, including reagent supply constraints, fragmented logistical workflows, and a shortage of integrated bioinformatics pipelines, limit their full utilization (Yek et al., 2022). Consequently, many studies identified in this review relied on international service providers for critical analytical components. Strengthening local bioinformatics capacity and streamlining operational workflows will be essential to fully leverage existing infrastructure and reduce dependency on external providers. This pattern reflects a broader trend observed in emerging research ecosystems, where foundational scientific output often advances more rapidly than translational and implementation capacity (Reyes et al., 2025).

Nevertheless, the region is well-positioned to narrow this gap. Growing investment in healthcare innovation and biotechnology, together with projections indicating rapid expansion of the GCC human microbiome market driven by precision medicine and preventive healthcare demand^3^, demonstrate increasing opportunities for the commercialization of microbiome-based solutions. Bridging discovery to application will require strengthened public-private partnerships that connect academic research institutions with biotechnology companies, healthcare providers, and industry stakeholders. In this context, innovation hubs, startup incubators, and technology transfer programs emerging across the GCC countries can play a critical role in accelerating microbiome-based product development, supporting microbiome-focused startups, and facilitating regulatory and market pathways. National biotechnology strategies, alongside targeted investment in healthy aging and longevity research such as initiatives supported by the Hevolution Foundation (Aldriwesh et al., 2026), further position the region to advance microbiome-based diagnostics, therapeutics, and precision health applications. Continued alignment among research institutions, healthcare systems, investors, and biotechnology sectors will be essential to fully realize potential opportunities and to translate microbiome research into tangible societal and economic benefits across the GCC countries.

### 4.2 Gaps in human, animal, and environmental microbiome research in GCC countries

In the GCC countries, microbiome research remains unevenly distributed across human, animal, and environmental domains, with important gaps that limit a comprehensive understanding of microbial ecosystems and their relevance to regional health and sustainability. Within the human microbiome domain, we found that research has focused predominantly on the gut (52%) and oral cavity (28%), with particular attention to metabolic disorders such as obesity and diabetes, which place a substantial health burden on GCC populations (Figure 5; Fan and Pedersen, 2021; Hou et al., 2022). This emphasis reflects regional public health priorities, as the GCC countries rank among the highest globally for type 2 diabetes and obesity, with age-adjusted diabetes prevalence reaching 25% in Kuwait and 19% in Saudi Arabia (IDF Diabetes Atlas, 2021), and obesity prevalence exceeding 35% in four GCC countries, led by Qatar, Kuwait, and Saudi Arabia^4^ However, other body sites have remained comparatively underexplored. Respiratory, urogenital, and skin microbiomes collectively accounted for less than 20% of human microbiome investigations in the region. This narrow research focus restricts a comprehensive understanding of host-microbiome interactions across multiple organ systems and environmental contexts. Such gaps are particularly relevant to regional health challenges, including respiratory conditions associated with frequent dust exposure (Gusareva et al., 2019), dermatological disorders influenced by extreme climatic conditions (Swaney and Kalan, 2021), and reproductive health concerns linked to urogenital microbiome dynamics (Ravel et al., 2011).

The limited number of animal microbiome studies (11%) represents a gap in the region's capacity to address zoonotic disease risks and support food security. Among the small number of available studies, camels were the most frequently investigated species (Figure 6A), reflecting their cultural and economic importance in the GCC countries. However, no studies examined the microbiomes of commonly consumed livestock such as cattle, sheep, or goats—animals that are central to regional food systems and may serve as reservoirs of antimicrobial resistance and zoonotic pathogens (Van Boeckel et al., 2015). This gap is particularly important given the region's history of zoonotic disease emergence. Middle East respiratory syndrome coronavirus (MERS-CoV), which continues to cause sporadic outbreaks, originated in dromedary camels and demonstrates the importance of understanding microbial dynamics at the animal-human interface (Azhar et al., 2014). The limited integration of animal microbiome research into broader health and surveillance frameworks reduces the region's preparedness to detect and respond to emerging zoonotic threats and reinforces the need to expand microbiome investigations in livestock and other animal populations.

Environmental microbiome research, while relatively more established (40%), has focused predominantly on soil and desert ecosystems (Figure 6B), with comparatively limited attention to the built environment, indoor air quality, and food production systems. Given the unique environmental conditions of the GCC countries including extreme temperatures, high salinity, rapid urbanization, and frequent dust storms, understanding how these factors shape environmental microbiomes and influence potential transmission pathways to human and animal hosts represents an important research priority.

### 4.3 Regional disparities and global positioning of microbiome research in GCC countries

The distribution of microbiome research output across the GCC countries reveals clear disparities in research activity (Figure 2B). These variations appear to be influenced by differences in geographic scale, population size, and the strength of research infrastructure. For example, Saudi Arabia, the largest GCC country by land area and population, maintains an extensive network of research-intensive institutions, including King Abdulaziz City for Science and Technology (KACST) and King Abdullah International Medical Research Center (KAIMRC), and supports major biomedical initiatives such as the Saudi Human Genome Program (Al-Farsi et al., 2021; Uddin and Alharbi, 2023). In comparison, Qatar demonstrates relatively high research output despite its smaller land area and population, likely reflecting substantial investment by organizations such as Qatar Foundation and Hamad Medical Corporation (Chouchane et al., 2011; Al-Farsi et al., 2021). In contrast, Oman and Bahrain produced more limited research output, which may reflect smaller research sectors, fewer specialized institutions, and lower health research expenditure relative to gross domestic product (GDP; Al-Farsi et al., 2021). Although such variation is expected, countries with lower research output face health, agricultural, and environmental challenges similar to neighboring states but currently possess a more limited microbiome evidence base to inform policy development and public health strategies. Beyond differences in national productivity, analysis of the included studies revealed limited regional collaboration among the GCC countries. Most investigations were conducted within single-country settings, with relatively few multicountry or jointly coordinated studies despite shared environmental conditions, dietary patterns, and interconnected ecosystems across the region. The absence of strong intra-regional collaboration restricts opportunities for large-scale cohort studies, comparative analyses, and harmonized data generation necessary for advancing microbiome science within a One Health framework.

These regional limitations become more apparent when the GCC microbiome research is considered within the broader global landscape. Microbiome science has rapidly expanded since the launch of large initiatives such as the HMP and the Metagenomics of the Human Intestinal Tract consortium (Turnbaugh et al., 2007; Peterson et al., 2009; Qin et al., 2010; Proctor et al., 2019), with more than 225,000 microbiome-related publications indexed in PubMed as of February 2026. Despite this global growth, microbiome datasets remain geographically imbalanced; more than 71% of publicly available human microbiome samples originate from Europe and North America and other high-income regions, leaving populations from the Middle East substantially underrepresented in global reference databases (Abdill et al., 2022).

Within this context, microbiome research in the GCC countries remains at a relatively early stage of development yet holds considerable untapped potential. The 110 studies identified in this review represent a modest contribution to global microbiome literature despite the GCC hosting nearly 60 million individuals with distinct genetic backgrounds, dietary transitions, and environmental exposures that are insufficiently captured in existing Western-centric datasets. At the same time, the region possesses distinctive characteristics across human, animal, and environmental domains that position it as an important setting for advancing microbiome science (Table 4). These include diverse and understudied populations, unique animal reservoirs, extreme desert and marine ecosystems, and rapidly evolving urban environments. Increasing regional investment in health, biotechnology, and healthspan research further strengthens the foundation for future expansion. Collectively, these factors position the GCC countries as an important context for generating novel insights into host-microbiome interactions across diverse populations and environmental conditions while contributing to improved global representation in microbiome research.

**Table 4A:** Human-related factors and microbiome R&D opportunities in the GCC countries.

Unique factor	Key information	R&D opportunities
Genetic uniqueness	• High consanguinity rates (~50%) across GCC populations (Bener and Mohammad, 2017) • Pronounced founder effects: 2,908 founder variants identified, including 224 novel gene-disease associations; 34% of variants absent from gnomAD (AlAbdi et al., 2025) • Established national genomics initiatives (the Saudi Genome Program^a^, Qatar Genome Program^b^, UAE Omics Center of Excellence^c^) enabling large-scale population sequencing	• Characterize population-specific microbiome signatures associated with high homozygosity and founder variants • Identify host genetic determinants that modulate microbiome composition and function • Integrate host genomic and microbiome datasets to stratify disease risk and inform precision medicine approaches in GCC populations
Nutrition transition	• Shift from traditional diets (e.g., dates, camel milk, and fermented foods) to Westernized dietary patterns high in ultra-processed foods and sugars • Obesity represents a major public health challenge in GCC countries, several of which rank among the highest globally, with prevalence exceeding 30% in Kuwait, Saudi Arabia, Qatar, and the UAE (Hammami and Alsnag, 2026)	• Characterize microbiome compositional and functional shifts associated with the transition from traditional to Western dietary patterns • Examine associations between diet-induced microbiome alterations and obesity, diabetes, and cardiometabolic risk markers in GCC populations • Develop and validate microbiome-informed, culturally tailored dietary interventions to prevent and manage metabolic disorders
Metabolic disease burden	• GCC countries rank among the nations with the highest global prevalence of diabetes and obesity (Phelps et al., 2024; Genitsaridi et al., 2026) • Rapid epidemiological transition and lifestyle changes contributing to early-onset metabolic disorders (Arredouani, 2021)	• Investigate gut-metabolic axis mechanisms in high-risk populations • Identify microbiome signatures associated with obesity in GCC cohorts • Develop and validate microbiome-based diagnostics and targeted interventions tailored to regional populations
The Hajj pilgrimage	• Hajj is the world's largest annual mass gathering • Approximately 2–3 million pilgrims annually from more than 60 countries^d^ • Documented acquisition and transmission of antimicrobial resistance (AMR) organisms during pilgrimage due to high-density, short-duration global population mixing (Alreeme et al., 2022)	• Quantify cross-border microbial and AMR transmission patterns • Develop and implement microbiome-informed AMR surveillance systems during mass gatherings • Model microbial transmission networks to inform global pandemic preparedness strategies
Quality of life programs	• Hevolution Foundation: among the world's largest funders of healthspan research, with an annual budget of approximately USD 1 billion^e^; microbiome research identified as a priority area in aging science • Saudi Vision 2030: national life expectancy target increase from 74 to 80 years and emphasis on preventive healthcare transformation^f^ • Regional prioritization of healthy aging and chronic disease prevention	• Establish dedicated microbiome-aging research programs aligned with national longevity strategies • Leverage large-scale healthspan funding to accelerate translational microbiome research in age-related diseases • Develop and evaluate microbiome-based preventive interventions to improve healthy lifespan in GCC populations

^a^https://www.vision2030.gov.sa/en/explore/projects/the-saudi-genome-program.

^b^https://www.ga4gh.org/.

^c^https://www.mediaoffice.abudhabi/en/topic/omics-centre-of-excellence/.

^d^https://www.vision2030.gov.sa/media/0hwbcwev/pep-annual-report-2023-english.pdf.

^e^https://hevolution.com/about.

^f^https://www.vision2030.gov.sa/en/media/articles/saudi-arabia-s-bet-on-healthspan-not-just-lifespan.

**Table 4B:** Animal-related factors and microbiome R&D opportunities in the GCC region.

Unique factor	Key information	R&D opportunities
Dromedary camel	• MERS-CoV reservoir confirmed by WHO^a^ • Camel milk contains diverse lactic acid bacteria strains with reported probiotic potential (Sheikh et al., 2024) • Approximately 1.8 million camels in Saudi Arabia^b^, with substantial regional livestock distribution	• Characterize camel microbiome composition across respiratory, gastrointestinal, and milk niches • Map microbial and viral transmission interfaces between camels and humans • Isolate and functionally evaluate probiotic candidate strains from camel-associated microbiota • Integrate camel microbiome data into regional One Health surveillance frameworks
Arabian horse	• Culturally and economically significant equine breed in the region • Fecal microbiota composition varies with diet, housing, and management practices (Curadi et al., 2025) • Comparatively understudied relative to Western horse breeds	• Characterize gut microbiome diversity across varied husbandry and care practices • Assess links between microbial profiles and equine health and performance • Compare Arabian horse microbiomes with those of other breeds to identify unique signatures • Integrate findings into animal health monitoring and breeding programs
Falcons	• Culturally significant raptors • Gut microbiota shaped by diet and enriched in *Salmonella* (Ahmad et al., 2024)	• Develop microbiome-informed strategies to improve falcon health • Conduct zoonotic pathogen surveillance • Explore wildlife microbiome ecology and diversity

^a^https://www.who.int/news-room/fact-sheets/detail/middle-east-respiratory-syndrome-coronavirus-(mers-cov).

^b^https://saudipedia.com/en/how-many-camels-are-there-in-saudi-arabia.

**Table 4C:** Environmental-related factors and microbiome R&D opportunities in the GCC region.

Specific environment	Unique factor	Key information	R&D opportunities
Marine microbiome	Red Sea coral	• Corals in the Red Sea exhibit exceptional thermal tolerance relative to many global reef systems (Raimundo et al., 2024) • Distinct coral-associated microbial communities adapted to high temperature and salinity (Raimundo et al., 2024) • King Abdullah University of Science and Technology Coral Probiotic Village initiative supporting microbiome-based reef research^a^	• Characterize microbial communities associated with thermally tolerant corals • Elucidate microbiome-mediated mechanisms underlying heat resilience • Develop and test probiotic consortia to enhance coral survival and reef restoration under climate stress
	Red Sea brine pools	• Deep-sea brine pools in the Red Sea, including recent discoveries near NEOM (Purkis et al., 2022) • Extremophilic microorganisms thriving at high temperature and high salinity (Ahmed et al., 2022) • Production of bioactive compounds and extremozymes with reported biotechnological potential (Ahmed et al., 2022)	• Isolate and characterize extremophilic microbes and their metabolic pathways • Identify and optimize novel enzymes and bioactive molecules for industrial and biomedical applications • Examine brine pool microorganisms as models for life under extreme planetary conditions
	Arabian Gulf marine	• First shotgun metagenomic survey conducted in a Kuwait marine protected area (Fakhraldeen et al., 2024) • Chronic exposure to extreme salinity and high sea surface temperatures • Distinct and highly adapted marine microbial communities (Fakhraldeen et al., 2024)	• Characterize microbial diversity and functional gene profiles in high-salinity marine environments • Identify and evaluate extremophile-derived enzymes and metabolites for industrial and biomedical applications • Advance marine bioprospecting pipelines for sustainable biotechnology development in the GCC
	Aquaculture	• Rapid growth of aquaculture production across GCC countries • Emerging fish microbiome research initiatives in Oman^b^ • National prioritization of food security and sustainable sea food production	• Characterize gut and environmental microbiomes in farmed fish species • Develop and validate probiotic and microbiome-modulating feed formulations to enhance fish health and productivity • Integrate microbiome monitoring into sustainable aquaculture management frameworks
Agriculture microbiome	Date palm ecosystem	• More than 30 million date palms cultivated in Saudi Arabia^c^ • Distinct root-associated (rhizosphere) and fruit microbiomes (Abumaali et al., 2023b) • Long-standing, endemic agricultural system adapted to arid environments	• Characterize rhizosphere and fruit-associated microbial communities across date palm cultivars • Investigate plant-microbiome interactions that enhance drought tolerance and crop resilience • Identify microbiome-derived compounds with potential functional food applications • Develop microbiome-informed strategies to support sustainable date palm agriculture
	Livestock production	• Extensive sheep, goat, and cattle farming across GCC countries • Active cross-border livestock trade within and beyond the region • Strategic prioritization of livestock production for national food security^d^	• Characterize gut and rumen microbiomes across major livestock species • Assess the impact of feed composition on microbial function and productivity • Develop and evaluate microbiome-informed feed strategies to enhance growth efficiency and reduce methane emissions • Strengthen microbiome-based pathogen surveillance to improve food safety and biosecurity
	Green initiatives	• Saudi Green Initiative^e^ • UAE Net Zero 2050 commitment to achieve net-zero emissions by 2050^f^	• Characterize soil microbiome dynamics in large-scale afforestation and land restoration projects • Quantify microbial contributions to soil carbon sequestration under arid conditions • Develop and evaluate microbiome-based strategies to enhance ecosystem restoration and climate resilience • Integrate environmental microbiome monitoring into national sustainability frameworks

^a^https://marinemicrobiomeslab.kaust.edu.sa/rsrc-coral-probiotics-village.

^b^https://faolex.fao.org/docs/pdf/oma214204E.pdf.

^c^https://ncpd.gov.sa/en.

^d^https://programs.grc.net/wp-content/uploads/2024/09/GCC-Agriculture-Sector-Outlook_2.pdf.

^e^https://www.sgi.gov.sa/.

^f^https://aesg.com/wp-content/uploads/2022/01/UAE-Net-Zero-2050.pdf.

### 4.4 Unique One Health-related microbiome research opportunities in GCC countries

Perhaps the most striking finding of this review is the absence of studies adopting a One Health framework that simultaneously examines human, animal, and environmental microbiomes. This gap extends beyond a scientific limitation and represents a barrier to understanding the complex microbial exchanges that influence health outcomes across interconnected ecosystems in the region (Muhummed et al., 2025; Winkler et al., 2025). Several characteristics of the GCC countries make this gap particularly significant (Table 4). Dromedary camels, recognized as reservoirs for MERS-CoV, remain insufficiently investigated from a microbiome perspective despite their close contact with human populations and their importance in regional food systems through milk and meat production (Dudas et al., 2018). In addition, the Hajj pilgrimage generates large-scale annual population movements that may facilitate intercontinental microbial exchange, yet the microbiome implications of these mass gatherings remain largely unexplored (Sarkar et al., 2024). Environmental factors also warrant closer attention. Seasonal dust storms transport microbial communities across national borders and may influence both environmental and respiratory microbiomes, but their role in shaping host-microbiome interactions in the region has not been systematically examined (Rees et al., 2021). Without integrated research approaches capable of tracing microbial dynamics across human, animal, and environmental domains, opportunities to better understand zoonotic disease emergence, food-associated microbiomes, and environmentally driven health risks will remain limited. Advancing coordinated, cross-domain microbiome investigations will therefore be important for strengthening regional preparedness, improving food safety and nutritional research, and clarifying how environmental exposures shape disease risk across GCC populations.

The need for integrating microbiome research within a One Health framework extends beyond disease prevention to include food security, antimicrobial resistance, and climate adaptation (Hernando-Amado et al., 2019; Proctor et al., 2025). Livestock microbiomes influence animal productivity and methane emissions, soil microbiomes play a central role in determining agricultural sustainability in arid environments, and marine microbiomes affect fisheries and aquaculture outcomes. These domains are interconnected through shared water resources, food systems, and patterns of human-animal-environment interaction. A coordinated One Health-oriented microbiome research strategy would enable the GCC countries to address these interconnected challenges more systematically. Such an approach could support the development of evidence-based interventions that enhance food system resilience, monitor and mitigate antimicrobial resistance, and improve environmental sustainability. In doing so, the GCC countries could contribute knowledge relevant not only locally but also to other arid and rapidly developing regions facing comparable ecological and public health pressures.

### 4.5 Strategic framework for advancing One Health microbiome research in GCC countries

**Figure 8:**
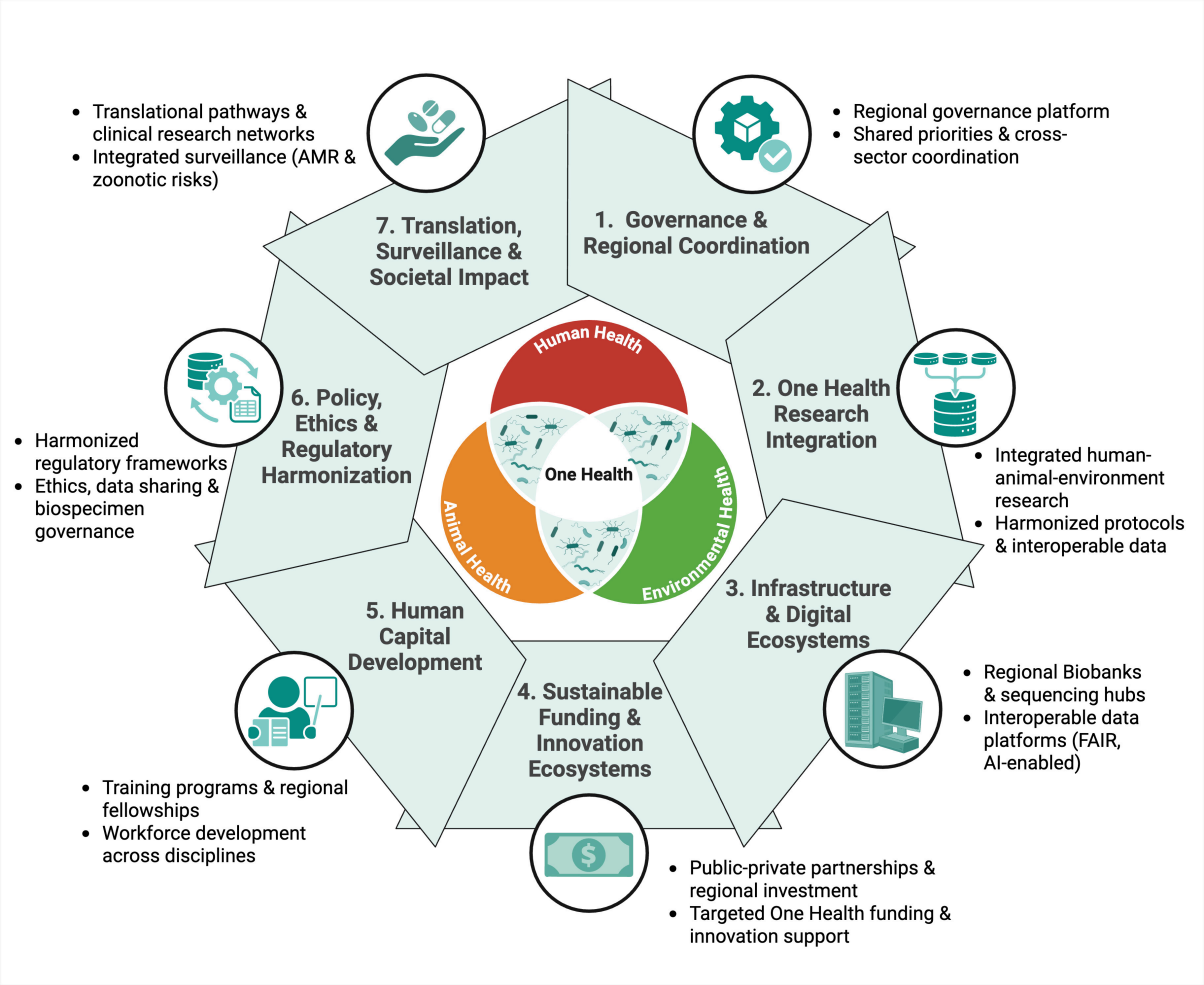
Strategic framework for advancing One Health microbiome research in GCC countries. Conceptual illustration of a coordinated GCC One Health microbiome framework supported by six strategic pillars designed to strengthen regional collaboration and enable the translation of microbiome research into health, agricultural, and environmental applications across GCC countries. The figure was created in BioRender. Aldriwesh, M. (2026) https://BioRender.com/9k66b1z.

Based on the findings of this review, advancing microbiome research across the GCC countries requires a coordinated strategic framework capable of addressing current fragmentation while leveraging the region's growing scientific investment, institutional capacity, and existing research infrastructure. Drawing on lessons from international initiatives such as the World Microbiome Partnership (Proctor et al., 2025), HMP (Peterson et al., 2009), the US National Microbiome Initiative (McCarthy, 2016), and the UK Microbiome Strategic Roadmap (Hashmi et al., 2022), we propose an integrated and implementation-oriented framework structured around interconnected strategic pillars designed to strengthen coordination, enable cross-sectoral research, and accelerate translation within a One Health framework (Figure 8).

#### 4.5.1 Governance and regional coordination

Progress toward a coordinated GCC microbiome ecosystem will depend on establishing regional governance mechanisms that integrate human health, animal health, agricultural, and environmental sectors. Such coordination would enable the definition of shared research priorities aligned with regional health, food security, and sustainability goals while promoting efficient allocation of resources across the GCC countries. A regional governance structure could also facilitate alignment with international One Health initiatives and strengthen collaboration with global microbiome consortia through scientific advisory engagement and coordinated policy direction. Existing regional bodies may provide a practical foundation for such coordination. For example, the Gulf Health Council (GHC)^5^, established in 1976, functions as an intergovernmental body responsible for coordinating health policy across the GCC countries and could serve as a platform to support and operationalize regional microbiome governance efforts.

#### 4.5.2 One Health research integration

Adopting the One Health framework as the central organizing principle is essential to overcome the current separation between human, animal, and environmental microbiome investigations in the GCC countries. Integrated research programs should deliberately examine microbial exchange across interconnected systems, particularly within regionally relevant contexts such as camel-human interfaces, mass gatherings including the Hajj pilgrimage, and environmental exposure pathways linked to dust storms, marine ecosystems, and arid agricultural environments. Further, expanding the scope of sampling to include underrepresented body sites, diverse animal hosts, and varied environmental reservoirs will be critical to achieving a more comprehensive understanding of microbiome dynamics in the region. Harmonization of study designs and analytical protocols across institutions would further enable cross-country comparisons, regional meta-analyses, and generation of interoperable datasets. These efforts align with existing international health frameworks. The GCC countries are States Parties to the International Health Regulations (World Health Organization, 2016), which emphasize surveillance, preparedness, and response to public health threats. Integrating microbiome research within such frameworks could support surveillance of zoonotic diseases and antimicrobial resistance, thereby strengthening regional health security and preparedness.

#### 4.5.3 Infrastructure and digital ecosystems

Sustained advancement of microbiome research will require shared infrastructure capable of supporting large-scale collaborative studies beyond the capacity of individual institutions. The development of coordinated regional Biobank networks operating under standardized collection, processing, and storage protocols would enable longitudinal investigations and facilitate international collaboration. Complementary investment in regional bioinformatics hubs and interoperable data platforms aligned with FAIR (Findable, Accessible, Interoperable, and Reusable) principles would support secure data exchange and analytical standardization. As microbiome datasets continue to increase in scale and complexity, integration of artificial intelligence and machine learning approaches alongside conventional analytical methods may enhance pattern recognition, enable cross-domain data integration, and support predictive modeling of antimicrobial resistance dynamics and microbial transmission within region-specific contexts. Such infrastructure could be further strengthened through the establishment of centralized sequencing hubs and a GCC-wide microbiome data repository supported by standardized metadata frameworks. Regional precedents for population-scale biological data infrastructure include the Saudi Human Genome Program, initiated by KACST in 2013 and subsequently aligned with Saudi Arabia's Vision 2030 strategic agenda^6^, and the Qatar Genome Program, established in 2015 under Qatar Foundation (Mbarek et al., 2022). These initiatives demonstrate the feasibility of coordinated, large-scale biological data generation and standardization within the region and provide transferable models for the development of microbiome-specific infrastructure and data ecosystems.

#### 4.5.4 Sustainable funding and innovation ecosystems

Long-term sustainability of microbiome research initiatives will depend on diversified funding structures that extend beyond traditional academic grants. Coordinated regional investment models incorporating public-private partnerships can accelerate innovation while reducing duplication of efforts across the GCC countries. The engagement of biotechnology industries, innovation incubators, and translational accelerators will be particularly important for supporting early-stage development and commercialization of microbiome-based diagnostics, therapeutics, agricultural technologies, and environmental applications. Such innovation ecosystems can facilitate the transition from discovery-driven research toward economically and societally impactful outcomes. National strategies such as Saudi Arabia's Vision 2030 Health Sector Transformation Program^7^, the UAE National Innovation Strategy^8^, and Qatar's National Research Strategy^9^ prioritize investment in biotechnology and translational health research, thereby creating opportunities for microbiome initiatives to develop and expand within the region. In this context, targeted regional funding calls for One Health microbiome projects could further incentivize cross-country and cross-sector collaboration.

#### 4.5.5 Human capital development

The expansion of microbiome science across the GCC countries ultimately depends on sustained investment in human capital. Establishing graduate and postgraduate training programs in microbiome science within regional universities, supported by harmonized curricula and cross-institutional mobility, would strengthen scientific capacity and promote interdisciplinary expertise. Joint training programs and regional fellowship schemes could further support the mobility of researchers across the GCC institutions. Professional development opportunities targeting clinicians, veterinarians, environmental scientists, and data specialists will further enable integration of microbiome approaches into research and practice. The GCC Health Council's established systems for continuing medical education and health professional mobility across the GCC countries provide a foundation for developing microbiome-focused training programs and cross-institutional fellowships (see text footnote 5). In parallel, public and stakeholder engagement initiatives remain important for increasing awareness among policymakers and healthcare systems regarding the potential value of microbiome research.

#### 4.5.6 Policy, ethics, and regulatory harmonization

Effective translation of microbiome discoveries requires regulatory environments capable of supporting innovation while ensuring ethical oversight and biosafety. Harmonization of regulatory frameworks across the GCC countries governing microbiome-based therapeutics, biospecimen sharing, data governance, and emerging artificial intelligence applications would facilitate cross-border collaboration and responsible commercialization. Alignment of ethical standards will also strengthen public trust and enable participation in multinational microbiome initiatives. The GCC Standardization Organization (GSO)^10^ has developed technical standards across key areas, including laboratory diagnostics, pharmaceutical products, and food safety testing within GCC countries. These standards provide a regulatory foundation that can support the development of microbiome-based therapeutics and biospecimen governance.

#### 4.5.7 Translation, surveillance, and societal impact

Advancing microbiome research from discovery to implementation will require dedicated translational pathways linking research institutions, healthcare systems, agriculture, and environmental management sectors. Regional clinical research networks capable of evaluating microbiome-based interventions in GCC populations can generate locally relevant evidence to guide implementation. Regional surveillance systems, including GCC infection control initiatives and antimicrobial resistance monitoring frameworks implemented across GCC countries, demonstrate regional capacity for coordinated health surveillance across national boundaries (Balkhy et al., 2016; Light et al., 2022). These systems could provide a platform for integrating microbiome-informed monitoring approaches. Integration of microbiome surveillance into One Health monitoring systems may further strengthen preparedness for antimicrobial resistance and emerging zoonotic risks. Progress should ultimately be evaluated not only through academic outputs but through measurable improvements in disease prevention, food system resilience, and environmental sustainability.

The convergence of scientific opportunity, regional investment, and policy commitment across the GCC countries provides a timely foundation for coordinated action. Collectively, this framework outlines strategic pathway through which the GCC countries can transition from fragmented research activity toward an integrated One Health microbiome ecosystem capable of generating globally relevant scientific and translational impact.

## 5 Conclusion

Microbiome research in the GCC countries is expanding rapidly but remains fragmented across human, animal, and environmental domains, with uneven regional distribution. The limited adoption of integrated One Health frameworks constrains a comprehensive understanding of microbial interactions relevant to health and ecosystem resilience. Nevertheless, the GCC possesses unique demographic and environmental characteristics, alongside increasing investment in health and biotechnology, creating strong opportunities to advance microbiome science. Addressing current gaps will require coordinated regional action through strengthened governance, integrated One Health research, shared infrastructure, workforce development, and effective translation pathways. The strategic framework proposed in this review provides a practical pathway toward a coordinated GCC microbiome research ecosystem capable of generating meaningful scientific and societal impact at both regional and global levels.

## Data Availability

The original contributions presented in the study are included in the article/Supplementary material, further inquiries can be directed to the corresponding author.

## Footnotes

1 Accessed from https://www.connectedpapers.com/.

2 Accessed from https://www.nhlbi.nih.gov/health-topics/study-quality-assessment-tools.

3 Accessed from https://mobilityforesights.com/product/gcc-human-microbiome-market.

4 Accessed from https://data.who.int/indicators/i/C6262EC/BEFA58B.

5 Accessed from https://www.ghc.sa/en/.

6 Accessed from https://www.vision2030.gov.sa/en/explore/projects/the-saudi-genome-program.

7 Accessed from https://www.vision2030.gov.sa/en/explore/programs/health-sector-transformation-program.

8 Accessed from https://u.ae/en/about-the-uae/strategies-initiatives-and-awards/strategies-plans-and-visions/strategies-plans-and-visions-untill-2021/national-innovation-strategy.

9 Accessed from https://qrdi.org.qa/en-US/.

10 Accessed from https://www.gso.org.sa/en/standards/.
