# Supplementary material for: Microbiome and One Health in GCC countries: current status, research gaps, and future directions
Source: Front Microbiol. 2026 May 29;17:1821688. doi: 10.3389/fmicb.2026.1821688 (PMC13260183; doi:10.3389/fmicb.2026.1821688)
Supplement: Supplementary file 1 [file Table_1.docx]

Supplementary Material

# Supplementary Tables

**Supplementary Table 1A. Data extraction tool for human microbiome studies conducted in the Gulf Cooperation Council (GCC) countries.**

| **Study no.** | **Citation and study title** | **Publication year** | **Country, sample origin** | **Study design** | **Disease focus** | **Sample type** | **Sample size and sex** | **Age group, years** | **Study period** |
| --- | --- | --- | --- | --- | --- | --- | --- | --- | --- |
|  |  |  |  |  |  |  |  |  |  |

**Supplementary Table 1B. Data extraction tool for human microbiome studies conducted in the Gulf Cooperation Council (GCC) countries.**

| **Study no.** | **Citation and study title** | **Method** | **Platform** | **Sponsor** | **Summary of findings** |
| --- | --- | --- | --- | --- | --- |
|  |  |  |  |  |  |

**Supplementary Table 2A. Data extraction tool for animal microbiome studies conducted in the Gulf Cooperation Council (GCC) countries.**

| **Study no.** | **Citation and study title** | **Publication year** | **Country, sample origin** | **Study design** | **Sample type** | **Sample size** | **Age** | **Study period** |
| --- | --- | --- | --- | --- | --- | --- | --- | --- |
|  |  |  |  |  |  |  |  |  |

**Supplementary Table 2B. Data extraction tool for animal microbiome studies conducted in the Gulf Cooperation Council (GCC) countries.**

| **Study no.** | **Citation and study title** | **Method** | **Platform** | **Sponsor** | **Summary of findings** |
| --- | --- | --- | --- | --- | --- |
|  |  |  |  |  |  |

**Supplementary Table 3A. Data extraction tool environmental microbiome studies conducted in the Gulf Cooperation Council (GCC) countries.**

| **Study no.** | **Citation and study title** | **Publication year** | **Country** | **Study design** | **Sample type** | **Sample size** | **Study period** |
| --- | --- | --- | --- | --- | --- | --- | --- |
|  |  |  |  |  |  |  |  |

**Supplementary Table 3B. Data extraction tool environmental microbiome studies conducted in the Gulf Cooperation Council (GCC) countries.**

| **Study no.** | **Citation and study title** | **Method** | **Platform** | **Sponsor** | **Summary of findings** |
| --- | --- | --- | --- | --- | --- |
|  |  |  |  |  |  |
